# Perinatal Specimens of *Saurolophus angustirostris* (Dinosauria: Hadrosauridae), from the Upper Cretaceous of Mongolia

**DOI:** 10.1371/journal.pone.0138806

**Published:** 2015-10-14

**Authors:** Leonard Dewaele, Khishigjav Tsogtbaatar, Rinchen Barsbold, Géraldine Garcia, Koen Stein, François Escuillié, Pascal Godefroit

**Affiliations:** 1 Directorate 'Earth and History of Life', Royal Belgian Institute of Natural Sciences, rue Vautier 29, B-1000, Brussels, Belgium; 2 Research Unit Palaeontology, Department Geology and Soil Sciences, Ghent University, Krijgslaan 281, 9000, Ghent, Belgium; 3 Institute of Paleontology and Geology, Mongolian Academy of Sciences, Ulaanbaatar, 210–351, Mongolia; 4 Université de Poitiers, IPHEP, UMR CNRS 7262, 6 rue M. Brunet, 86073, Poitiers cedex 9, France; 5 Earth System Science, AMGC, Vrije Universiteit Brussel, Pleinlaan 2, 1050 Brussels, Belgium; 6 Eldonia, 9 Avenue des Portes Occitanes, 3800, Gannat, France; Raymond M. Alf Museum of Paleontology, UNITED STATES

## Abstract

**Background:**

The Late Cretaceous Nemegt Formation, Gobi Desert, Mongolia has already yielded abundant and complete skeletons of the hadrosaur *Saurolophus angustirostris*, from half-grown to adult individuals.

**Methodology/Principal Findings:**

Herein we describe perinatal specimens of *Saurolophus angustirostris*, associated with fragmentary eggshell fragments. The skull length of these babies is around 5% that of the largest known *S*. *angustirostris* specimens, so these specimens document the earliest development stages of this giant hadrosaur and bridge a large hiatus in our knowledge of the ontogeny of *S*. *angustirostris*.

**Conclusions/Significance:**

The studied specimens are likely part of a nest originally located on a riverbank point bar. The perinatal specimens were buried by sediment carried by the river current presumably during the wet summer season. Perinatal bones already displayed diagnostic characters for *Saurolophus angustirostris*, including premaxillae with a strongly reflected oral margin and upturned premaxillary body in lateral aspect. The absence of a supracranial crest and unfused halves of the cervical neural arches characterize the earliest stages in the ontogeny of *S*. *angustirostris*. The eggshell fragments associated with the perinatal individuals can be referred to the *Spheroolithus* oogenus and closely resemble those found in older formations (e.g. Barun Goyot Fm in Mongolia) or associated with more basal hadrosauroids (*Bactrosaurus*-*Gilmoreosaurus* in the Iren Dabasu Fm, Inner Mongolia, China). This observation suggests that the egg microstructure was similar in basal hadrosauroids and more advanced saurolophines.

**Competing Interests:**

One of the authors (FE) is employed by the commercial organization Eldonia. Eldonia provided support in the form of a salary for FE, but did not have any additional role or influence in the study design, data collection and analysis, decision to publish, or preparation of the manuscript and it does not alter the authors’ adherence to all the PLoS ONE policies on sharing data and materials.

## Introduction

The 'Dragon’s Tomb' dinosaur locality was discovered in 1947, in the Nemegt Formation (late Campanian / early Maastrichtian, Late Cretaceous) of the Gobi Desert, by the Russian Palaeontological Expedition to Mongolia’s Gobi Desert, led by I. A. Efremov. The bone bed at this site has yielded numerous articulated skeletons of the giant saurolophine hadrosaurid *Saurolophus angustirostris* Rozhdestvensky, 1952 [1]. This dinosaur is particularly abundant in the whole Nemegt Formation, comprising approximately 20% of all vertebrate fossils [2] found. The skull lengths of the known *S*. *angustirostris* specimens extend from about 437 mm (MgD-1/159) up to 1220 mm (PIN 551/357), thus already covering a wide array of ontogenetic stages, from juveniles to adult individuals. However, embryonic and neonatal remains have not been described to date.

Because of the quantity and quality of dinosaur skeletons from Mongolia, the whole of the Nemegt Formation has become a favorite target for poachers, and untold numbers of *Saurolophus* specimens are now in private hands around the world or have been destroyed in the process of poaching.

Here, we describe and discuss specimen MPC-D100/764: an exceptional block of perinatal specimens of *Saurolophus angustirostris*, with associated eggshell fragments, from the Nemegt Formation. The skull length of these babies is around 5% of that of the largest known *S*. *angustirostris* specimens, so they document the earliest development stages of this giant hadrosaur and bridge a large gap in our knowledge of the ontogeny of *Saurolophus angustirostris*.

## Materials and Methods

Originally poached from the Nemegt Formation, specimen MPC-D100/764 resided in a private collection for an unknown amount of time. Neither the exact geographic nor stratigraphic origin is known. The specimen has been treated chemically in order to solidify the matrix. This is evidenced by both stains on the specimen and a differential mechanical resistance between the outer surface and the interior. In 2013, the specimen was transferred to the Royal Belgian Institute of Natural Sciences (RBINS), Brussels, through the French company Eldonia. Subsequent negotiations between the RBINS, Eldonia and Mongolian authorities led to the official return of the specimen to Mongolia, where it is now catalogued as specimen MPC-D100/764.

One of the femora and one dorsal vertebral centrum with associated neural arch were sampled for osteohistological analysis. The femur was sectioned at the Research Unit Mineralogy and Petrology, Department Geology and Soil Sciences at Ghent University, Belgium, whereas the vertebral elements were sectioned in the Service de Paléontologie Animale et Humaine, Departement of Geology at the University of Liège, Belgium. The longitudinal section of the femur was machine-ground to a thickness of 30 μm, the vertebral sections were ground to 50 μm. Sections were studied using a polarized light microscope Olympus BH-2 (femur) and a Nikon LV 100 (vertebra) polarized light microscope. Pictures of the thin sections were taken with a ColorView I (femur) and a QImaging MP5.0 (vertebra) digital microscope camera. The femur was selected because its poor state of preservation prevented the accurate description of the bone and because it could easily be removed from the block without damaging any other elements. However, because of its poor state of preservation and post-mortem displacement, the exact orientation of the longitudinal thin section cannot be elucidated. The dorsal centrum was selected because it was easily accessible, and would allow comparison with other published dorsal vertebrae of embryonic dinosaurs.

During further preparation of MPC-D100/764 in Belgium, two fragmentary eggshells were found closely associated with the skeletal material, suggesting that the individuals were still enclosed in their eggs when they were covered by sediments, or that they died shortly after hatching. The description of the outer and inner surfaces of the eggshell fragments is based on macroscopic examination and by using a binocular microscope, while observation of their crystallographic ultrastructure was made by scanning electron microscopy (SEM). Because there are only two small eggshell fragments associated with MPC-D100/764, the study of the eggshell microstructure through thin sections, requiring a large number of sections in three dimensions has not been attempted: this process would have destroyed the eggshell fragments for future research. Nomenclature and terminology used for the parataxonomy is adopted from Mikhailov [3].

### Ethics statements

The fossil described in this paper was poached from the Nemegt Formation and sold in Japan, then in Europe. It was located in a private collection by one of us (FE), and then donated to the RBINS. Negotiations with the Ministry of Culture, Sports and Tourism of Mongolia led to the official restitution of this specimen to the Institute of Paleontology and Geology of the Mongolian Academy of Sciences at Ulaan Baatar, where it is currently housed (MPC-D100/764). The Ministry of Culture, Sports and Tourism of Mongolia subsequently provided the requested authorizations for studying and publishing this specimen. Therefore, the described study complies with all relevant regulations.

### Sedimentological and taphonomic contexts

Because this fossil was not discovered first-hand by the authors of the present paper, and because it was not accompanied by precise locality information, the exact geological context of this fossil remains unknown. However, close examination of the sediments surrounding the fossils had been carried out to try to elucidate the depositional environment of the *Saurolophus* babies.

**Fig 1:**
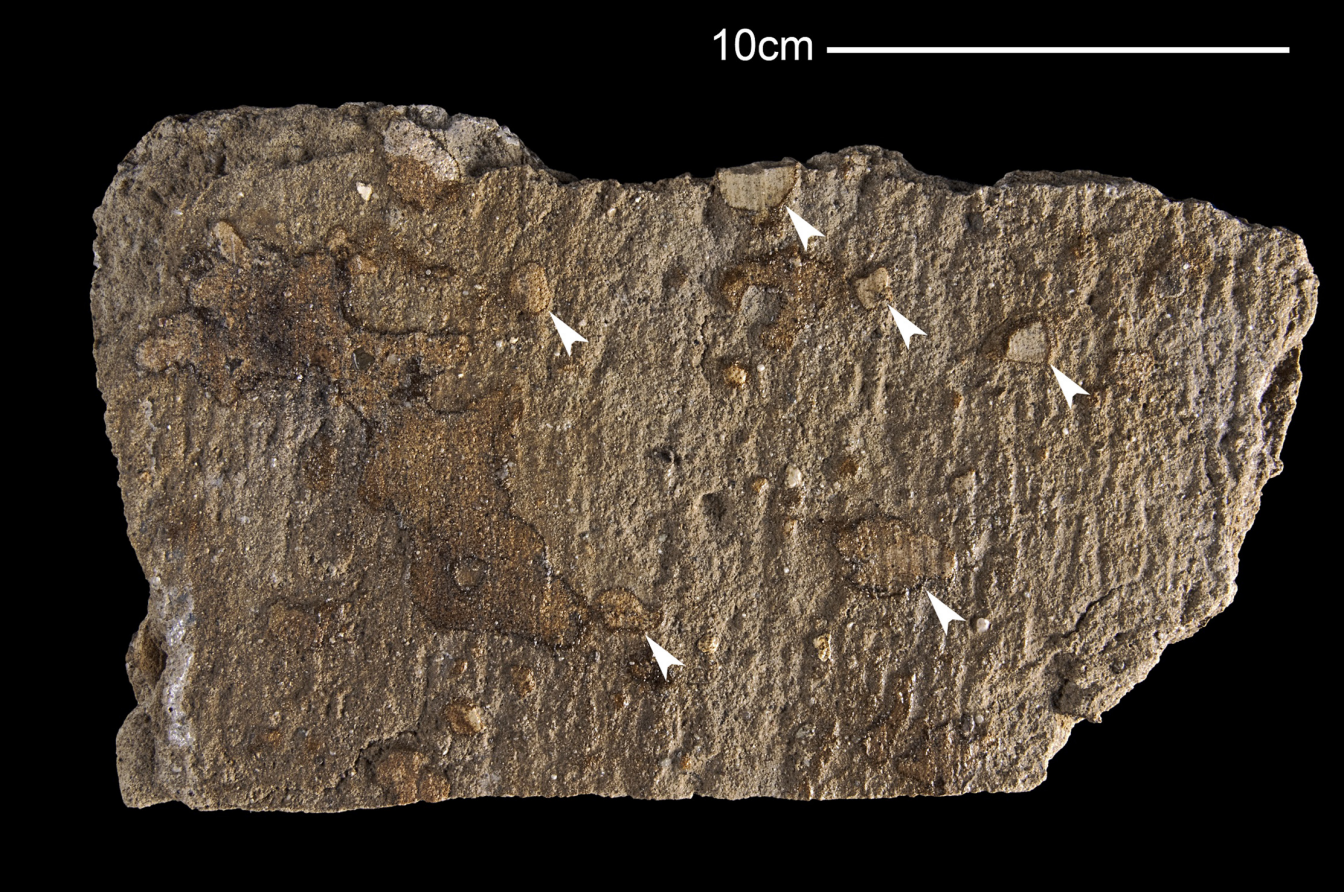
Sediments surrounding the *Saurolophus angustirostris* perinatal specimens MPC-D100/764. Intraformational pebbles set apart from matrix by differences in color and relief of sawing plane. Intraformational pebbles indicated by white arrows.

The sediment around the fossils is composed of poorly consolidated yellowish-gray sandstone. Sedimentary structures could not be identified, partly because of the small size of the observable area (order of a few square decimeters, see Fig 1) and because post-discovery fractures partly obscured natural structural features. Nonetheless, a significant number of intraformational pebbles are preserved (Fig 1). These pebbles differ from the surrounding sediment by their paler color and in being more resistant to physical weathering.

**Fig 2:**
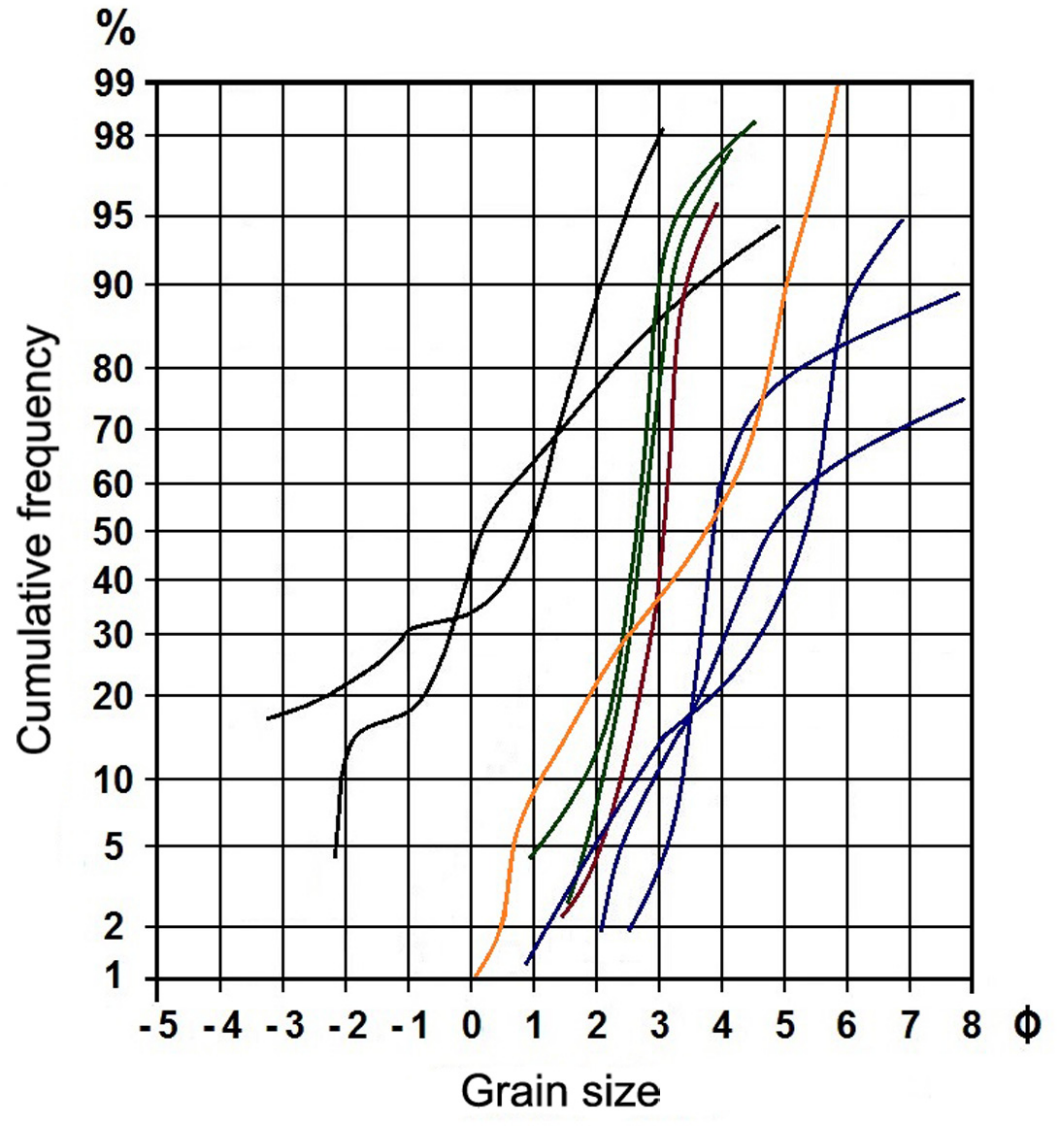
Cumulative frequency plot of different sediment samples of the Nemegt Formation. Grain size analysis of the current study (orange) plotted against measurement of Gradziński [4]. Sediments analyzed by Gradziński are: pebbly sandstone (black), sandstone with large-scale oblique stratification (green), sandstone with large-scale tabular oblique stratification (red), siltstone (blue).

Microscopically, the intraformational pebbles and the main part of the sandstone are identical in clastic sedimentary content: predominantly sand-sized quartz and feldspar grains with a minor percentage of lithic fragments, and silt-sized heavier minerals. They only differ in the absence or presence of calcite cement [4]. Comparisons with the grain size analyses of dinosaur-bearing sites from the Nemegt [4] Formation suggest that these heavy minerals consist primarily of epidote and titanite. The only difference is the sparitic cement present in the pebbles. The cumulative frequency plot of the grain sizes in the fossiliferous block does not fit well with any of the curves drawn by Gradziński [4] in the different sediment samples that he collected in the Nemegt Formation (Fig 2); this might be explained by the fact that Gradziński does not describe the parameters (e.g., long axis, short axis) he used to construct his cumulative frequency plots. In our analysis, the length of the B-axis of the largest inscribed ellipse of each grain is used. Unfortunately, indentations in the grains may cause the largest inscribed ellipse to shrink and the associated B-axis to be reduced. This may induce an overestimation of smaller grain size (i.e., see orange curve of Fig 2 offset to the right). The grain size measurements are listed as S1 File.

Gradziński [4] and Jerzykiewicz and Russell [5] have shown that the sediments of the Nemegt Formation were deposited in a flood plain environment intersected by meandering rivers and many braided tributaries. The paleoclimate of the Nemegt Formation was strongly seasonal, with dry and cold winters and wet and hot summers (e.g., [4, 6]). Because rainfall and runoff were limited in winter, most sediment transport and deposition probably occurred during the summer season. This paleoenvironment is expressed in the lithofacies of the Nemegt Formation. Coarse pebble and gravel-sized sediments were deposited at the river floor. When moving laterally during meandering, this basal gravel was covered by sandy sediments deposited either on the river bottom when the river current decreased or as foresets deposited at the point bars of the meandering rivers. When the lateral distance to the meandering river decreased, sediments only deposited in crevasse splays or during river flooding. This floodplain lithofacies predominantly consists of clay-sized sediments [4].

The perinatal specimens were clearly buried in sandy sediments; however, because sedimentary structures cannot be observed, a particular sedimentary setting (e.g. tabular or trough oblique stratification) cannot be assigned. The exceptional preservation of fragile bones and the presence of a partly articulated skeleton suggest that the specimens experienced little to no transport. Because humeri of three or four individuals are associated in MPC-D100/764, the perinatal individuals were probably close to each other when they died and most likely nest-bound, as also indicated by the presence of eggshell fragments close to the skeletons. They probably died within a relatively short time interval. This hypothesis is supported by the close association of the bones of the different individuals, their similar age profiles and the similar degree of weathering. The presence of a partly articulated skeleton associated with more disarticulated elements suggests that some individuals were in a more advanced state of decomposition than others. Consequently, it is probable that the more disarticulated, i.e., more decomposed, individuals died prior to the more articulated individuals. The inability of individuals to disperse from the already-deceased nestlings is indicative of the nest-bound nature of the individuals. Apart from the good preservation of fragile and fine bones, the association of eggshell fragments, small disarticulated bones and a partially articulated skeleton is difficult to explain by aggregation through river transport, because differential transport would disperse the differently sized eggshells and bones, rather than aggregating them.

For the nest to be buried by sand, it had to be located on a point bar. This contrasts with previous interpretations by Mikhailov et al. [7], that most eggs and eggshell fragments from the Nemegt Formation had been laid far from any riverbank. However, Mikhailov et al. [7] provide no evidence for this assumption. In fact, they argue that all eggs and eggshells from the Nemegt Formation have been recovered from rose or gray sands and sandstones, which have a fluviatile origin [4]. Because only two eggshell fragments have been discovered in association with the skeletal elements, it is likely that most other eggshell fragments either got buried slightly away from the block of MPC-D100/764 or got removed from the site prior to burial or that they were not recognized by the poachers during excavation.

### Osteological description of MPC-D100/764

**Fig 3:**
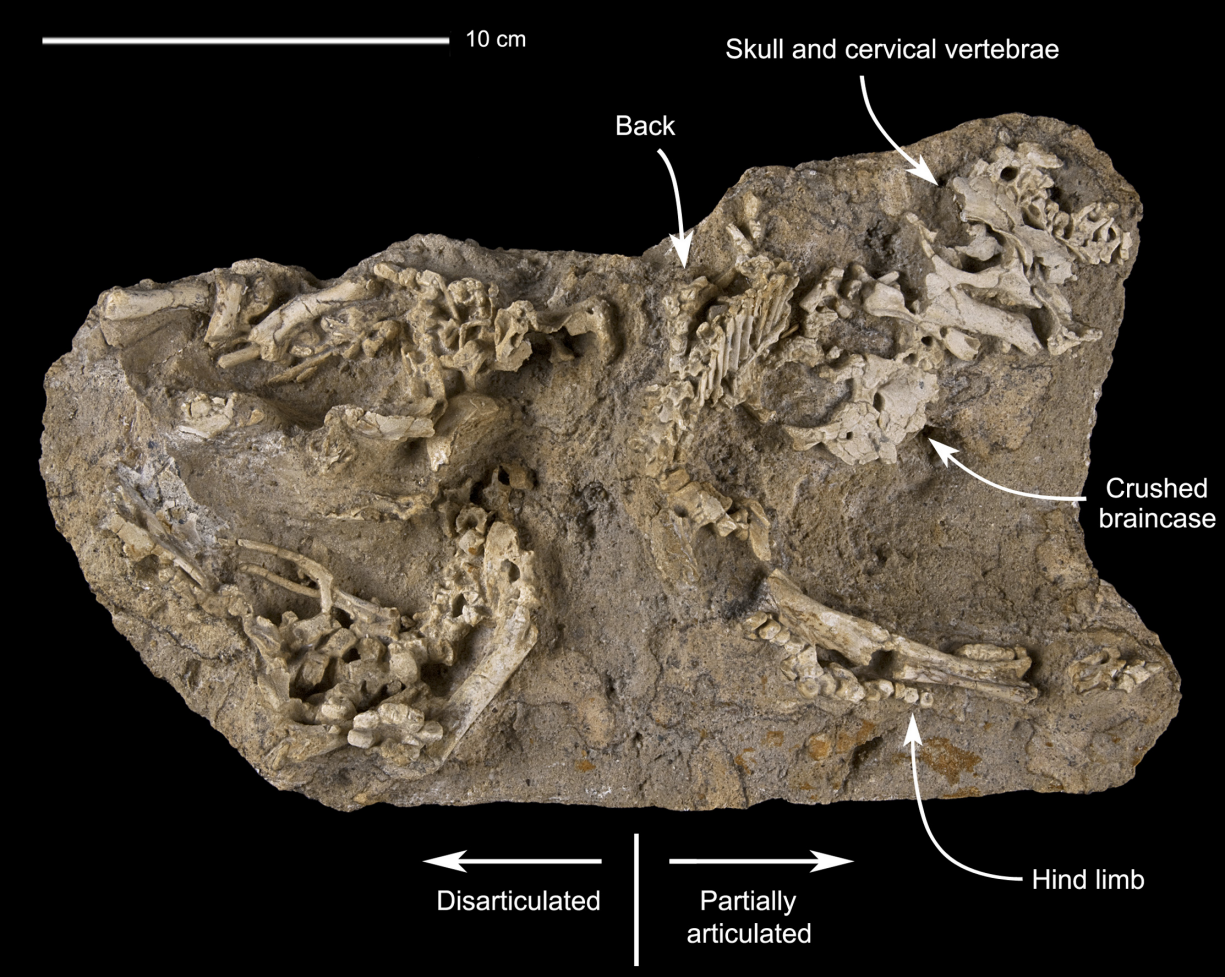
Perinatal specimens of *Saurolophus angustirostris* (MPC-D100/764). Bones on the right side of the block show a certain degree of articulation, whereas bones on the left are disarticulated.

MPC-D100/764 consists of cranial and postcranial remains of certainly three and possibly four individuals, based on the number of humeri (Fig 3). The right side of a partly articulated skeleton is preserved, including the skull, a series of cervical vertebrae, the partial thorax, sacral elements, the partial tail, and the right hindlimb; the matrix obscures the left side of this individual. Disarticulated bones of at least two or three additional individuals of similar size are also preserved. A concentration of disarticulated pectoral girdle and forelimb bones roughly coincides with the area for the missing left forelimb of the articulated skeleton, whereas a concentration of disarticulated pelvic girdle and hindlimb bones coincides with the area for the missing left hind limb of the articulated skeleton.

Length measurements of MPC-D100/764 are provided as S1 Table. Factors impeding straightforward length measurements are, first, the fractured nature of many bones and, second, the early ontogenetic stage of the bones in MPC-D100/764 in which bones are not necessarily already completely ossified. Hence, the length measurements should be considered with care.

### Cranium

#### Premaxilla (Fig 4)

The morphology of the premaxilla is autapomorphic for *Saurolophus angustirostris* [8]: the rostral and lateral borders of the body of the premaxilla are relatively thick and strongly upturned, giving the body of the premaxilla an overall depressed appearance. Given the overall good preservation of the anterior part of the skull, it is very unlikely that post-mortem crushing would have caused this reflection. This strongly upturned lip is unique among young saurolophines [9]. The caudolateral process is dorsoventrally flattened, as in older juvenile and adult specimens of *S*. *angustirostris* [8]. It laps on the rostrodorsal margin of the maxilla and extends to the middle of the rostrodorsal margin of the lacrimal. Three foramina are present along the posterior part of the caudolateral process and have not been described either in adult *Saurolophus* specimens [8], or in other perinatal hadrosaurids [10].

**Fig 4:**
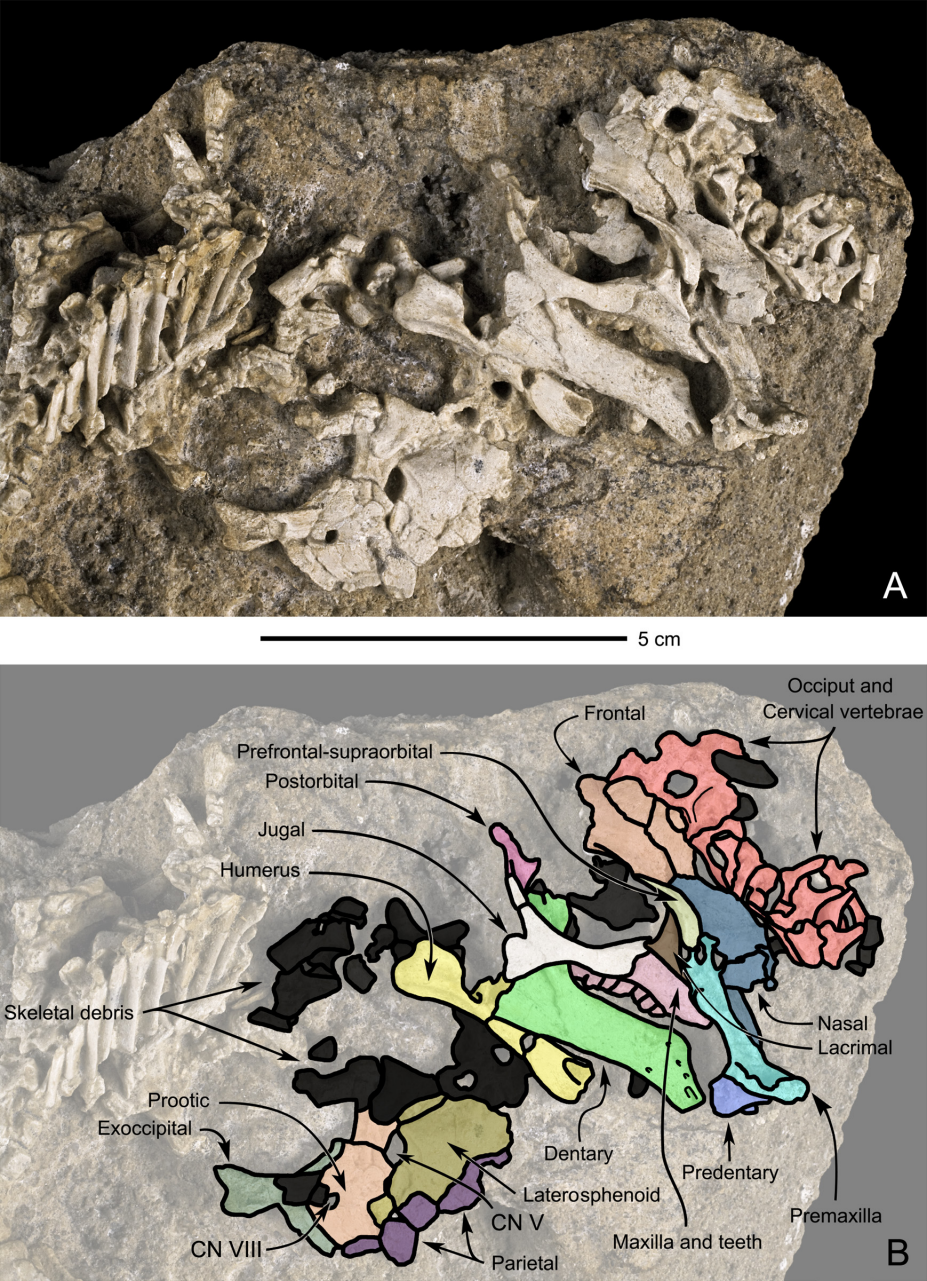
Perinatal specimens of *Saurolophus angustirostris* (MPC-D100/764). Articulated skull in right lateral view, partial braincase in left lateral view and cervical vertebrae. (A) without and (B) with bone identification. Color labels in (B) indicate: premaxilla (azure blue), maxilla and teeth (drab pink), nasal (navy blue), lacrimal (brown), jugal (white), prefrontal-supraorbital (olivine), postorbital (lilac), predentary (blue), dentary (green), frontal (orange); parietal (purple), laterosphenoid (drab yellow), prootic (apricot orange), exoccipital (moss green), occiput and cervical vertebrae (red), humerus (yellow), indeterminate material or skeletal debris (black). Cranial nerves V and VIII are indicated on (B).

The snout is proportionally short, when compared to larger specimens of *Saurolophus angustirostris* (see [11]). Neither the caudodorsal process of the premaxilla nor the external naris can be observed, because of post-mortem dislocation of the fractured nasal over the premaxilla.

#### Maxilla (Fig 4)

In lateral view, the maxilla is an obtuse isosceles triangle, as in other saurolophines (= “hadrosaurines”; [12]). The lateral side of the main body is flat, but its ventral margin is slightly inset. The ventral margin of the maxilla forms a broad platform with at least five, but possibly seven or more, teeth along its caudal three-fourths. The poor state of preservation of the teeth prevents their detailed description. It cannot be ascertained whether any denticles or tooth wear should have been present in the perinatal stage of *Saurolophus angustirostris*, as described in *Hypacrosaurus* perinatals [10]. Also, given the fact that the caudal portion of the maxilla and the maxillary tooth row is obscured by the jugal and deeply embedded in the matrix, it is impossible to ascertain whether the number of teeth is indeed limited to 5 or 7, or whether there are more teeth present. However, this number of teeth is in line with observations in juvenile and adult specimens of *S*. *angustirostris*: the juvenile ZPAL MgD-1/159 has 27 alveoli with up to four or five teeth per alveolus, while the adult PIN 551/358 has over 45 such alveoli [8]. Hence, the number of teeth increases with size in *S*. *angustirostris*. The number of teeth also increases with age in other hadrosaurids, such as *Hypacrosaurus*, in which perinatal specimens have also 5–7 maxilary teeth [10].

The rostral process of the maxilla underlays the posteroventral process of the premaxilla and the rostral process of the lacrimal. The entire dorsal process of the maxilla underlays the flattened rostral process of the jugal in lateral view.

#### Nasal (Fig 4)

One nasal can be identified on the articulated skull. The morphology of the nasal is usually diagnostic amongst saurolophines. However, the nasal of MPC-D100/764 is severely fractured, with fragments of the caudal part of the nasal slid over and covering its rostral part. Only the most rostral portion of the nasal is more or less in place and is only slightly displaced rostrally with respect to the premaxilla. Unlike larger specimens of *Saurolophus angustirostris*, which have a large external naris between the rostroventral process of the nasal and the premaxilla, no external naris can be observed in the articulated skull of the studied specimens. This suggests that the external naris was likely particularly small, an ontogenetically variable character already noticed by Bell [8].

There is no observable evidence of a nasal crest. This might be either broken off or due to the absence of a crest in perinatal *Saurolophus angustirostris*.

#### Lacrimal (Fig 4)

One right lacrimal is preserved, in lateral view. The lacrimal is roughly a right isosceles triangle with its caudal side forming a mediolaterally broad platform contributing to the rostral margin of the orbit. The rostral and dorsal processes are very slender and pointed. Given the absence of a noticeable external naris, it is impossible to test Bell’s [8] (p. 711) observation that “in adults, the anterior tip reaches a point level with and ventral to the posterior margin of the external narial opening, although in juveniles it is dorsal and posterior to the naris.” The body of the lacrimal laterally covers the jugal posteriorly and dorsal process of the maxilla rostrally. The lacrimal-maxilla contact appears particularly long, despite Bell’s [8] statement that the length of this contact is proportionally longer in larger specimens. However, it appears that the rostrolateral portion of the rostral process of the jugal is incompletely preserved and should have extended more rostrally. Also, given the dislocation of the prefrontal-supraorbital complex relative to the lacrimal, it is likely that, to some extent, the lacrimal had also undergone post-mortem dislocation rostrally. Hence, further description of contacts between the lacrimal and adjacent bones is hampered.

#### Jugal (Fig 4)

Although Bell [8] described the jugal bone as being W-shaped in lateral aspect in adult and juvenile specimens of both *Saurolophus osborni* and *Saurolophus angustirostris*, the jugal in the studied perinatal specimens is rather Y-shaped, with the postorbital process apparently rotated caudally, enlarging the ventral margin of the orbit. This is a common ontogenetically changing character present in perinatal and juvenile vertebrates in order to accommodate the relatively large eye balls. Among hadrosaurids, enlarged orbits are not only present in young *S*. *angustirostris*, but also in other young hadrosaurids, such as the lambeosaurine *Hypacrosaurus* [10]. The postorbital process is relatively thick, compared to older specimens of *S*. *angustirostris*. As already observed by Bell [8] in older specimens of *S*. *angustirostris*, its distal part is mediolaterally flattened for contact with the postorbital in the perinatal specimens of MPC-D100/764 as well.

Because of the small size and of the abrasion of the rostral process of the jugal in our specimen, we could not unambiguously observe a rostral spur on the rostral process of the jugal, as described by Bell [8] in subadult and adult specimens. The rostrodorsal tip of the anterior process is inserted between the maxilla and the posterior tip of the lacrimal.

#### Prefrontal-supraorbital complex (Fig 4)

The rostrodorsally curved prefrontal-supraorbital complex is mediolaterally flattened and participates in the rostrodorsal margin of the orbital rim. According to Bell [8], the suture between supraorbital I and supraorbital II should have been clearly visible in the early ontogenetic stages of *Saurolophus angustirostris*. However, no suture is present in the prefrontal-supraorbital complex of MPC-D100/764. Hence, both supraorbitals appear completely fused to each other and with the prefrontal, as in older ontogenetic stages and in *Hypacrosaurus* perinatals [10]. Caudomedially, the prefrontal-supraorbital complex contacts the frontal and rostromedially, the nasal. The prefrontal-supraorbital complex was obviously displaced rostrally, because it overlays the lacrimal in MPC-D100/764, although they are usually aligned, forming a smoothly curved anterodorsal margin of the orbital cavity [8].

#### Postorbital (Fig 4)

The postorbital is too incompletely preserved to be adequately described.

#### Frontal (Figs 4 and 5)

In the articulated skull, the frontal is located at the level of the dorsal margin of the orbital rim. It is excluded from the orbital rim by the postorbital and the supraorbitals, an important characteristic for lambeosaurines [10] and the saurolophine genera *Saurolophus* and *Prosaurolophus* (= Saurolophini) (see [8, 13]). The contact between the frontal and the prefrontal-supraorbital complex is difficult to observe and both appear fused. The frontal and the postorbital have a bridle contact, with the frontal being the mortise.

**Fig 5:**
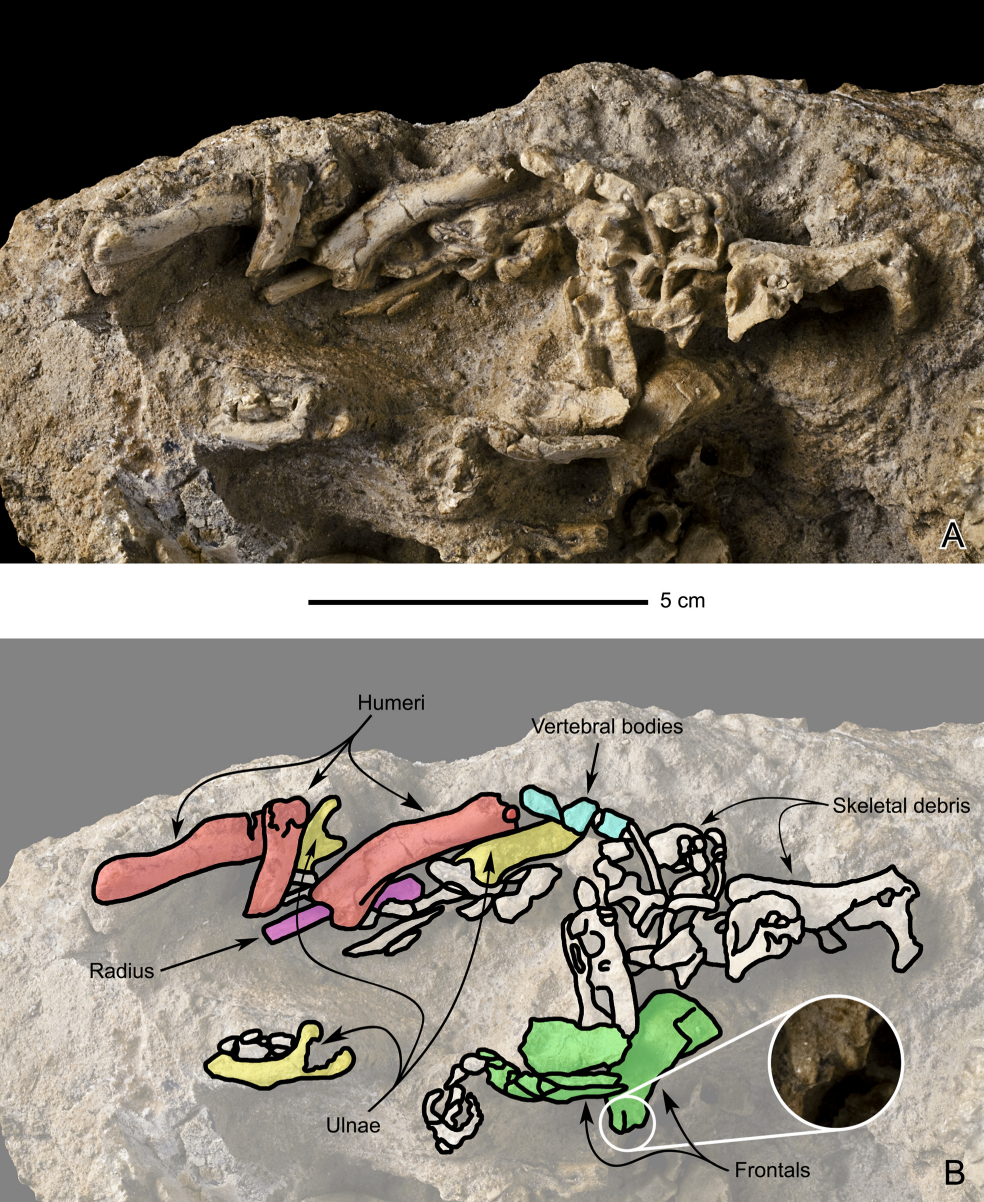
Perinatal specimens of *Saurolophus angustirostris* (MPC-D100/764). Disarticulated bones. (A) without and (B) with bone identification. Color labels in (B) indicate: humeri (red), radius (lilac), ulnae (yellow), centra of vertebrae (azure blue), frontals (green), indeterminate material or skeletal debris (white). Ambiguous incipient bifurcation of both frontal processes encircled in (B) and enlarged in inset image.

The thin-walled frontal is remarkably domed, which is characteristic for juvenile specimens within the known ontogeny of the saurolophines *Saurolophus angustirostris* [8, 11] and *Augustynolophus* [14], but also occurs in lambeosaurine ontogenetic series [9; 15–17]. Although the frontal remains domed in adult lambeosaurines and the hadrosauroid *Lophorhothon* [12], doming of the frontals decreases—and eventually disappears—in subadult and adult specimens of *S*. *angustirostris* [8, 11].

One of the disarticulated frontals shows poorly preserved rostroventral and caudodorsal processes; they are less developed than in larger specimens of *Saurolophus angustirostris* (e.g., [8]). Because these processes aid in supporting the supracranial crest in older individuals [8, 11], their poor development in MPC-D100/764 confirms that the supracranial crest was only very small and rudimentary in perinatal specimens of *S*. *angustirostris*. Both processes are very rudimentary and lack any distinct features. The caudodorsal process is longer than the rostroventral one.

#### Supraoccipital-opisthotic (Fig 4)

Located dorsal to the skull roof of the articulated individual, there is a series of cervical vertebrae and a complex that is interpreted as being the occiput. The poor state of preservation of the occiput, due to deformation, does not allow its detailed description

#### Braincase (Fig 4)

In MPC-D100/764, a severely crushed braincase is present in left lateral view. Although identifiable, bones are fragmentary and often broken off; and, hence, it is hard to pinpoint the exact location and shape of the contacts between adjacent elements. Identified bones include the prootic, the exoccipital, the laterosphenoid, and the parietal. In left lateral view, the prootic is a prominent bone, contributing significantly to the posterior part of the braincase. It borders the foramen of cranial nerve V posteriorly, and apparently encloses the foramen of the cranial nerve VIII. Unfortunately, the nature of one particular bone fragment is ambiguous and, hence, it is uncertain whether the prootic encloses cranial nerve VIII entirely or not.

The prootic contacts the laterosphenoid caudally, the (fragmentary) parietal caudally to caudodorsally, and the exoccipital body posteriorly to caudoventrally. A flange projects ventrally from the prootic, but, because of its poor state of preservation, it is not possible to draw conclusions on the exact shape of this flange.

At its rostral margin, the exoccipital covers the posterior edge of the prootic; and rostrodorsally the exoccipital abuts the most caudal edge of the parietal. The projecting caudal process is robust and represents the base of the paroccipital process. Distally, the base of this paroccipital process expands dorsoventrally. Yet, the natural edges of the exoccipital and the paroccipital process are severely damaged and their description is therefore tentative.

The laterosphenoid is relatively large and is located rostral to rostrodorsal of the prootic. The laterosphenoid is usually formed by three processes in hadrosaurids (see [18]); however, poor preservation has obscured these processes in MPC-D100/764. The postorbital process cannot be identified unambiguously, and only the base of the presumed basisphenoid process is visible. The blunt prootic process pinches out at the dorsal margin of the prootic. The left lateral surface of the laterosphenoid is flat and smooth. The laterosphenoid borders the foramen of cranial nerve V rostrally. Rostral to the laterosphenoid, an ambiguous suture separates the laterosphenoid from the parasphenoid. A fragmentary parietal contacts the dorsal and caudodorsal edges of the prootic and the rostrodorsal edge of the exoccipital. The parietal undoubtedly contacts the entire dorsal margin of the laterosphenoid, as has also been observed by Bell [8] in the juvenile PIN551/359.

#### Predentary (Fig 4)

The predentary is displaced and largely obscured by the premaxilla and the surrounding matrix. It has a horseshoe shape, as is typical in hadrosaurids [12]. Although not much can be observed, the predentary closely resembles that in the perinatal lambeosaurine *Hypacrosaurus* [10], with the lateral processes expanded dorsoventrally, in caudal direction. Due to its small size and limited exposure, neither denticles nor foramina could be observed and the predentary appears smooth.

#### Dentary (Fig 4)

The robust dentary is faintly sigmoidal in lateral view, with a straight body, a laterally- expanded posterior part and a rostral tip that tapers medially. Most of the ventral edge of the dentary is straight, but its rostral tip projects slightly more ventrally. Similarly, the buccal platform of the dentary is straight; yet, toward the rostral tip, the body of the dentary suddenly makes a sharp ventral dip, as also observed in the *Hypacrosaurus* perinatals [10]. The robust coronoid process is flattened mediolaterally and slightly inclined caudally, contrasting with the rostrally-inclined coronoid processes in larger specimens of *Saurolophus angustirostris* [11] and other adult hadrosaurids [12]. However, this character is clearly ontogenetic, as the coronoid process is also inclined caudally in *Maiasaura* (PG, pers. obs.) and *Hypacrosaurus* perinatals [10]. No dental teeth have been observed. Because the lingual side of all the available dentaries are embedded in the matrix. Five rostrally-opening neurovascular foramina are present on the rostral half of the labial surface of the dentary. The most anterior foramen—or mental foramen—is much larger than the others, as is usual in hadrosaurids [12].

### Axial skeleton

#### Cervical vertebrae (Fig 4)

**Fig 6:**
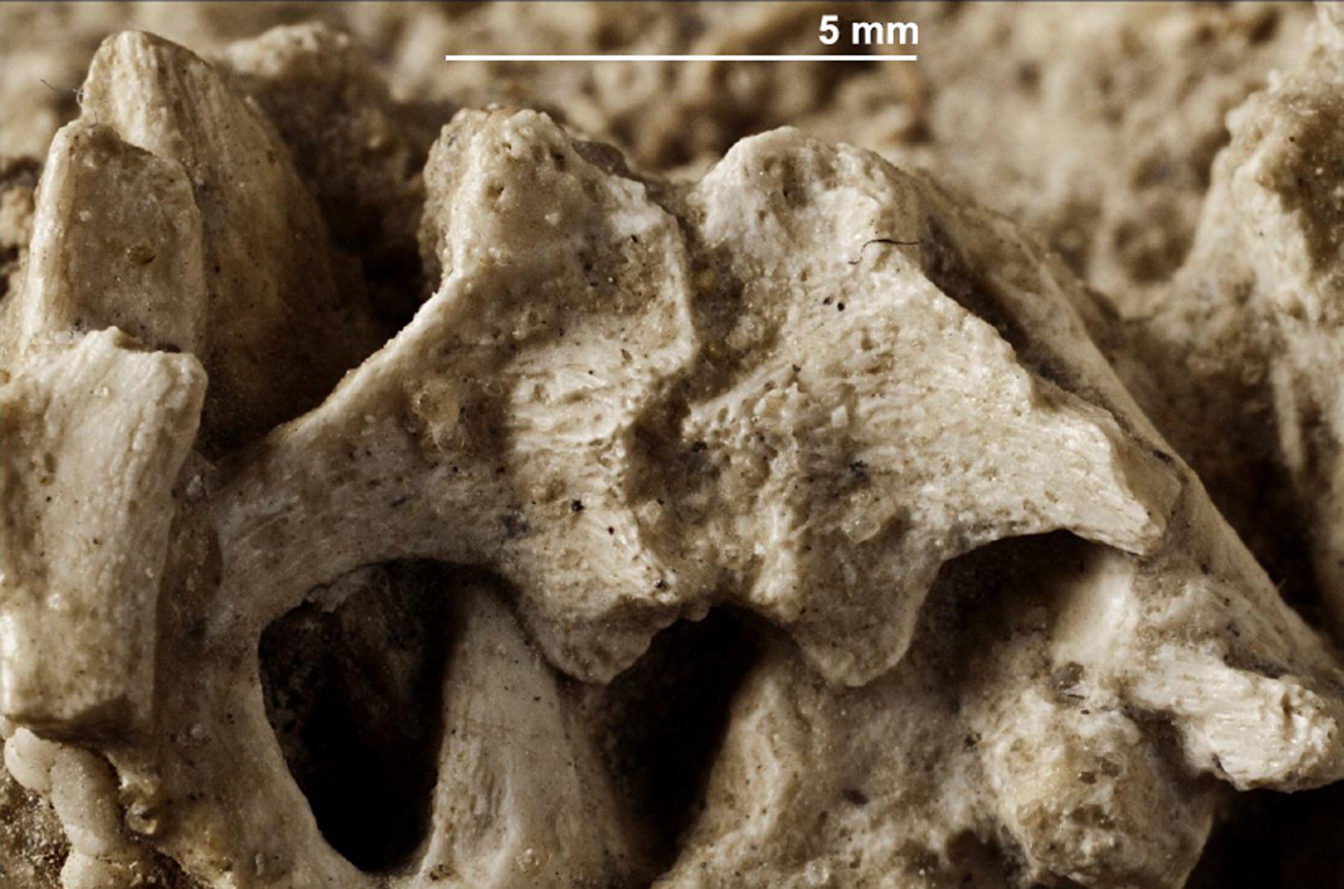
Perinatal specimens of *Saurolophus angustirostris* (MPC-D100/764). Close-up of a cervical vertebra, showing the suture dividing the neural arch. Note the fibrous and spongy bone texture.

A series of five or six cervical vertebrae is present near the articulated skull, representing about half of the number of cervical vertebrae in *Saurolophus angustirostris* [19]. The left and right halves of some of the delicate neural arches are clearly separated by a longitudinal suture (Fig 6). It is generally known that the neural processes at each lateral side of the spinal cord fuse during ontogeny to form a neural arch in living archosaurs [20, 21]; in ornithischian dinosaurs this character has already been observed in an embryonic *Camptosaurus* specimen [22].

The slender transverse processes project caudolaterally. Both the pre- and postzygapophyses are short and rounded, the prezygapophyses being slightly more robust. The articular facets of the pre- and postzygapophyses are slightly inclined, with the prezygapophyses facing slightly anteriorly and the postzygapophyses facing slightly posteriorly. Most of the neural spines are fragmentary; when preserved, they appear rather short and stout, as observed in adult specimens of both *Saurolophus angustirostris* and *Saurolophus osborni* [19]. Cervical centra are not visible.

#### Dorsal vertebrae (Fig 7)

A series of 9–10 dorsal vertebrae can be observed on the articulated skeleton. Because the dorsal vertebrae are all articulated and embedded in the matrix, the configuration of their articular surfaces of the vertebrae cannot be ascertained. In lateral view, they are slightly constricted in the middle. The ventral side has a prominent ventral keel. Both the pre- and postzygapophyses are short and rounded, the postzygapophyses being more slender. The transverse processes are robust and project caudally. The neural arches are not fused to the centra. The neural spines are relatively low and robust, projecting caudodorsally.

**Fig 7:**
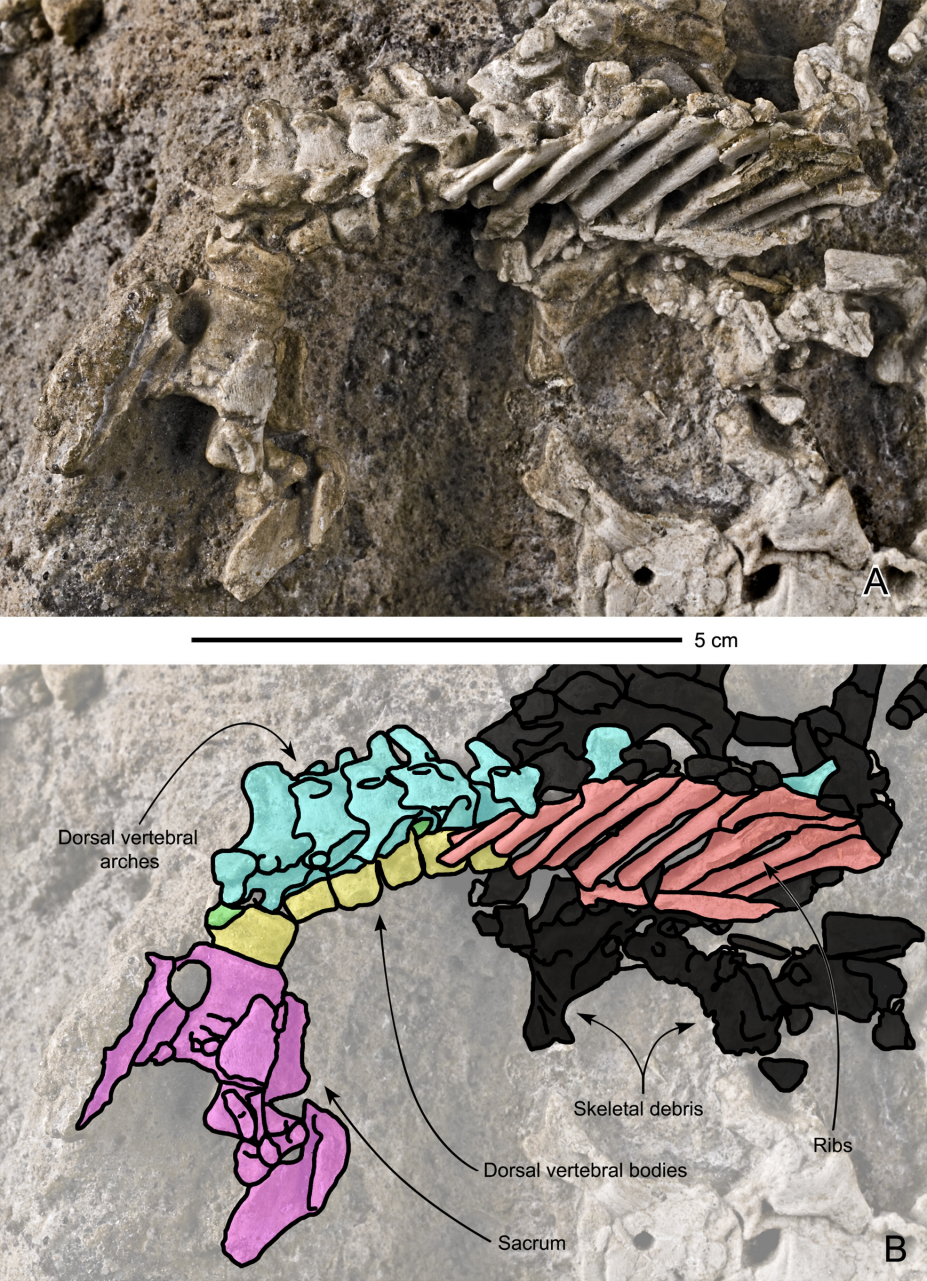
Perinatal specimens of *Saurolophus angustirostris* (MPC-D100/764). Articulated dorsal series and pelvis in right lateral view. (A) without and (B) with bone identification. Color labels in (B) indicate: dorsal ribs (red), arches of dorsal vertebrae (azure blue), centra of dorsal vertebrae (yellow), intercentra (green), sacrum and pelvis (lilac), indeterminate material or skeletal debris (black).

#### Dorsal ribs (Figs 7 and 8)

Lateral to the series of dorsal vertebrae, are nine right dorsal ribs still partly articulated; all are broken off near their capitulum, i.e., the capitulum and tuberculum are missing. Disarticulated dorsal ribs are also randomly present on the block. Among these disarticulated ribs, one rib has a very simple capitulum and tubercle preserved. As it is preserved, the tubercle is very rudimentary, being a featureless elevation over the rib. The capitulum is relatively long and slender cylindrical, yet simple. The ribs are slightly curved, suggesting that the thoracic cage was probably proportionally less wide than in larger specimens.

**Fig 8:**
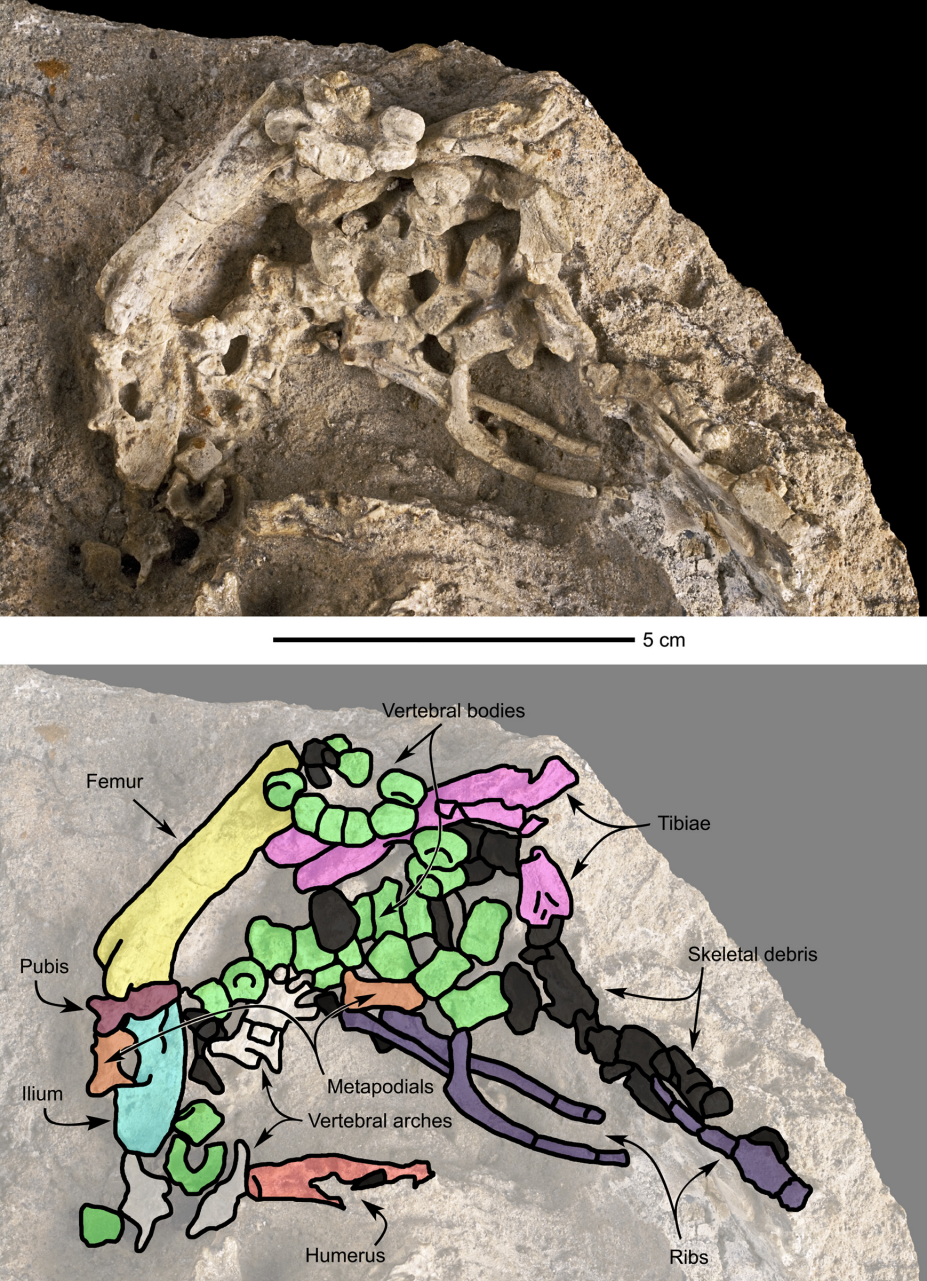
Perinatal specimens of *Saurolophus angustirostris* (MPC-D100/764). Disarticulated bones. (A) without and (B) with bone identification. Color labels in (B) indicate: centra of vertebrae (green), arches of vertebrae (white), ribs (purple), metapodials (orange), pubis (pubis), ilium (burgundy), femur (yellow), tibiae (lilac), humerus (red), indeterminate material or skeletal debris (black).

#### Caudal vertebrae (Fig 9)

A series of 14 caudal vertebrae are associated with the subcomplete specimen. Another series comprising seven caudals is also present on the opposite side of the tibia and fibula. All the observed centra are shortened and apparently amphicoelous, unlike adults and subadults of *Saurolophus angustirostris*, which have opisthocoelous proximal centra and amphiplatyan distal centra [19]. This probably reflects the incomplete ossification of the vertebrae (see [23]). The articular surfaces of the caudal vertebrae are subcircular. The neural arches are not preserved.

**Fig 9:**
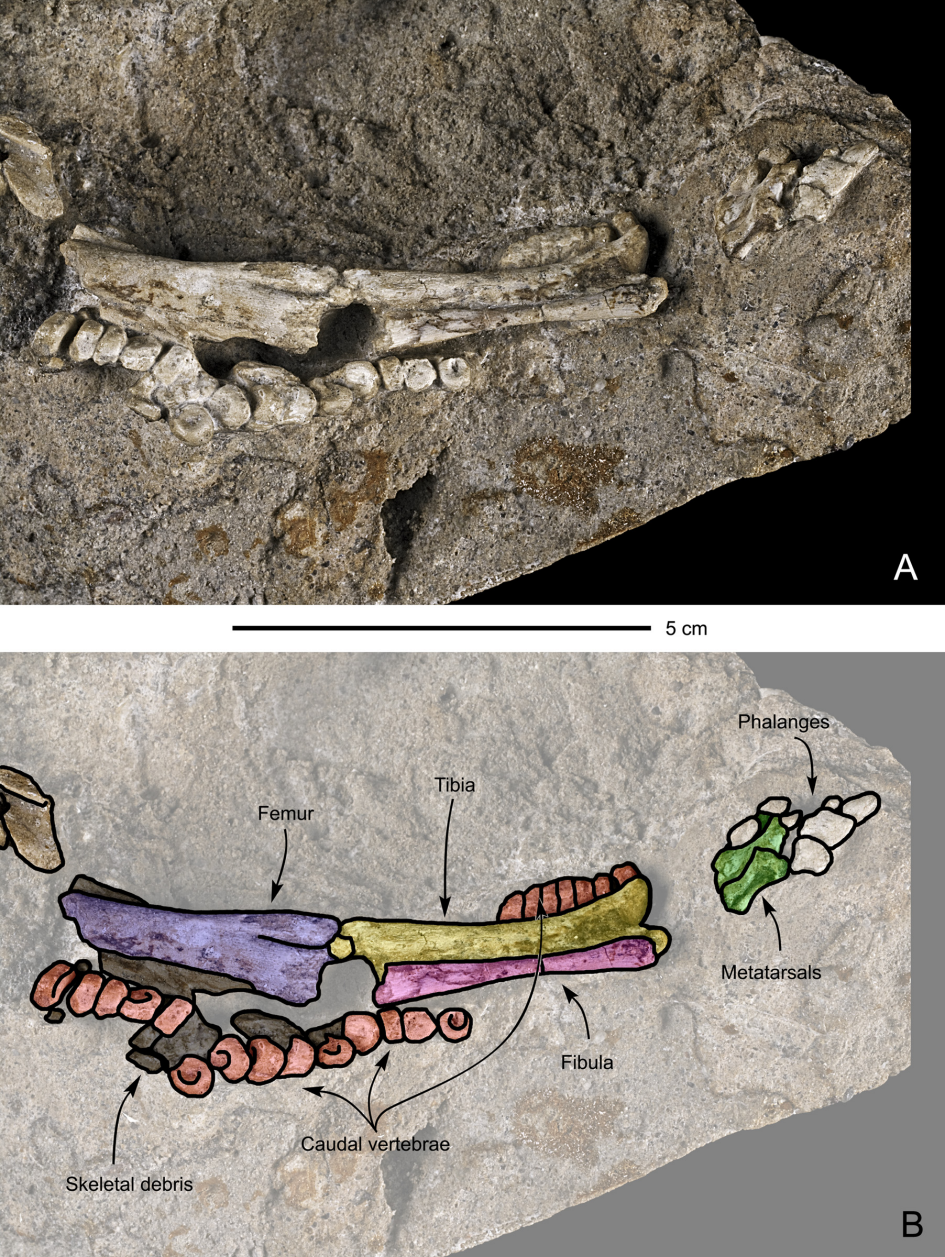
Perinatal specimens of *Saurolophus angustirostris* (MPC-D100/764). Right hindlimb and partial tail. (A) without and (B) with bone identification. Color labels in (B) indicate: caudal vertebrae (red), femur (yellow), fibula (lilac), tibia (azure blue), metatarsals (green), phalanges (white), indeterminate material or skeletal debris (black).

### Appendicular skeleton

#### Humerus (Figs 4 and 5)

Five humeri are identified. Four of them are grouped among the disarticulated bones, while the fifth lies near the skull of the partially articulated specimen. Of these five humeri, one could be identified as a left humerus and two as right ones. The two remaining humeri are too poorly preserved to be identified. This number of humeri allows us to state that bones of at least three and possibly four individuals are present.

The humerus is moderately elongate with a very robust deltopectoral crest extending along the proximal half of the humeral shaft, giving the humerus a sigmoidal appearance. Such a robust deltopectoral crest also characterises larger specimens of *Saurolophus angustirostris* [19] and, convergently, the whole lambeosaurine clade [12]. The articular head is prominent and rounded. The distal condyles are of similar size and weakly separated. Overall, the humerus is strongly similar to the juvenile and adult humerus of *S*. *angustirostris*. However, similar to other hadrosaurids (e.g. *Hypacrosaurus* [10]) the perinatal humerus is much more robust than the adult humerus.

#### Ulna (Fig 5)

Three ulnae (two left and one right) are present among the disarticulated bones. Unfortunately, only the proximal ends are visible. The olecranon process is particularly robust. According to Horner et al. [12], the ulna is straight in all Hadrosauridae, except in *Saurolophus angustirostris*. One of the preserved ulnae is distinctly convex posteriorly, thus showing some degree of curvature. This curvature is unlikely to be related to post-mortem processes, because other bones are not deformed.

#### Radius (Fig 5)

Only one identifiable radius is present among the disarticulated skeletal elements. Yet, its state of preservation, again, prevents its accurate description.

#### Ilium (Fig 8)

Only one ilium and one pubis have been unambiguously identified in the fossiliferous block. The rostral part of the ilium is largely obscured by an overlying femur and pubis. In general, the ilium appears robust and thickened. Ventrally, a semicircular recess marks the iliac contribution to the acetabulum. The apex of both pubic and ischiac peduncles are obscured by other bones. The postacetabular notch is only weakly marked. The postacetabular process is broken and the dorsal edge of the ilium is slightly convex. The lateral surface of the ilium is relatively flat.

#### Pubis (Fig 8)

The pubis is only poorly preserved and partly obscured by surrounding bones, so only the robust prepubic blade and the proximal parts of iliac and ischiac peduncles can be adequately described. Distally, the prepubic neck widens rapidly into an oval prepubic blade, slightly longer caudocranially than high dorsoventrally. This rapid widening of the prepubic neck is also characteristic for *Saurolophus angustirostris* adults, contrasting with the gently bowed dorsal and ventral edges of the proximal prepubis is *Saurolophus osborni* [24]. However, the widening of the prepubic neck is much stronger in the perinatal MPC-D100/764 than in larger *S*. *angustirostris* specimens. However, this observation might be explained by the significant damage and poor visibility of the pubis in MPC-D100/764.

The ilium and the pubis are proportionally more robust in the *Saurolophus angustirostris* perinatals than in larger specimens. The general morphology of both bones does not change significantly between the different ontogenetic stages in *Hypacrosaurus stebingeri* [10].

#### Femur (Figs 8 and 9)

Three femora are present in the fossiliferous block. One was sampled for microscopic investigation of the bone histology (see below). The femur is massive and straight. The proximal head is not preserved in either of the femora. The femoral shaft is subcircular in cross-section. The craniodistal part is very rudimentary and separated in two condyles by a narrow but deep extensor groove. This groove is offset, creating a relatively small medial condyle and a larger lateral condyle in anterior view. Both condyles are slightly compressed mediolaterally. In all present femora, sediment and other bones obscure the caudodistal part. No fourth trochanter could be discerned, the absence of which cannot be explained by the preservation of the specimens. The absence of the fourth trochanter is most probably related to the early ontogenetic stage of the specimens in which the bones are still very incomplete and rapidly growing. However, the fourth trochanter is well developed in *Hypacrosaurus stebingeri* perinatals [10].

#### Tibia (Figs 8 and 9)

Three tibiae appear to be present, of which only one has been identified unambiguously and suitable for description. The tibia is a robust bone and its distal half seems to be concave anteriorly. The cnemial crest appears poorly developed. The tibial shaft is subcircular. Distally, the tibia expands mediolaterally in two very robust conical malleoli, separated from each other by a deep sulcus. The external malleolus extends slightly more distally longer than the internal malleolus.

#### Fibula (Fig 9)

The right fibula of the sub-complete specimen is preserved, in articulation with the tibia. The fibula is a slender bone and is concave cranially. The central section of the fibular shaft is subcircular in cross-section. However, at its extremities, the fibula slightly expands. This expansion is more pronounced at the proximal end than at the distal end. The distal extremity of the fibula is rounded, whereas the proximal extremity is missing.

#### Pes (Fig 9)

The preserved part of the right pes of the subcomplete specimen consists of three metatarsals and five phalanges. The tarsals are absent, leaving a hiatus between the adjacent leg bones and The number of metatarsals of the articulated hind limb is in accordance with Horner et al. [12], stating that all hadrosaurs have only three digits (II, III and IV) at their pes. Given the 0-3-4-5-0 phalangal formula for hadrosaurs [12] seven phalanges are therefore missing in MPC-D100/764. The metatarsals are proportionally short and fairly robust. Metatarsal III is the largest one, slightly longer than metatarsal II and much longer than metatarsal IV. Furthermore, metatarsal III covers a major part of metatarsal II, hampering observations of metatarsal II. In dorsal view, all three metatarsals appear straight, contrasting with the diverging metatarsals II and IV in adult hadrosaurids [12]. The metatarsals are slightly constricted in their middle part. The distal ends of metatarsals II and III form two faint condyles, separated by a shallow intercondylar groove. On metatarsal IV, two small pits can be inferred, one on the lateral side and one on the medial side of the proximal extremity, separated by a small ridge. Although ambiguous, this structure may be considered as the articular facet for the two condyles of tarsal IV.

The preserved phalanges are very rudimentary. Their exact shapes could not be inferred, for each phalange morphologically differed from the others. These shape differences are, again, related to the rapid growth of the specimen. Any difference in growth rate between different phalanges or any difference in onset of phalanx growth may, at this ontogenetic stage, result in morphologically completely different ossified phalanges.

### Identification of the perinatal specimens

Two hadrosaurid taxa are known from the Nemegt Formation of Mongolia: *Saurolophus angustirostris* Rozhdestvensky, 1952 [1] and *Barsboldia sicinskii* Maryańska and Osmólska, 1981 [24] (see [8]). Both belong to the saurolophine clade [8, 25]. *Saurolophus angustirostris* is known from multiple individuals belonging to different ontogenetic stages (see [8, 11]), although *B*. *sicinskii* is only known from one incomplete postcranial skeleton [24, 25].

Identification as *Saurolophus angustirostris* is based on:

The exclusion of the frontals from the orbital margin; a synapomorphy separating the Saurolophini tribe (*Prosaurolophus–Saurolophus*) from other saurolophines [13].
The tripartite frontal; diagnostic for *Saurolophus*, contributing to the base of the nasal crest (Fig 5).
The upturned “lip” at the rostral and lateral edges of the body of the premaxilla; a generic autapomorphy shared by both *Saurolophus angustirostris* and *Saurolophus osborni* [8]. However, this lip is more strongly upturned in *S*. *angustirostris* than it is in *S*. *osborni*, giving the body of the premaxilla a strongly depressed look. Other researchers also consider the premaxillae of the North American saurolophine genera *Edmontosaurus*, *Gryposaurus* and *Prosaurolophus* strongly upturned [9, 26].

Besides these characteristics, other features can be used to identify the bones on this specimen as *Saurolophus angustirostris*. However, these remain ambiguous and should rather be used as a confirmation of the identification, e.g., the apparent curvature of ulna is characteristic for the species [12]. In our specimen, one of the ulnae exhibits a slight curvature. However, because this curvature is very slight and the ulna is largely embedded in the matrix, this character remains ambiguous.

## Bone Histology

Bone histological analysis has been performed on the aforementioned damaged femur, as well as a dorsal vertebral centrum with associated neural arches.

### Femur histology

**Fig 10:**
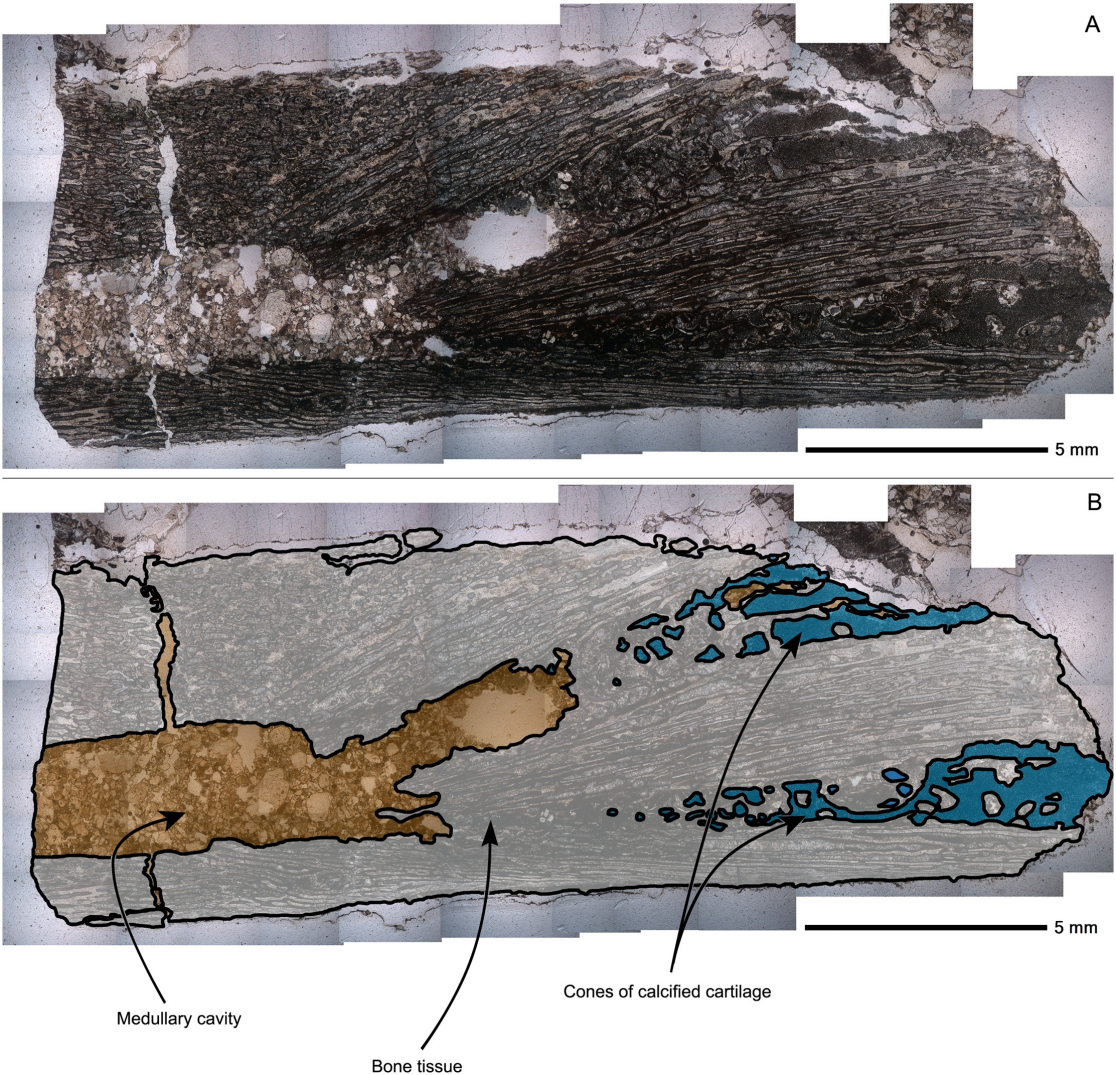
Perinatal specimens of *Saurolophus angustirostris* (MPC-D100/764). Composite image of the longitudinal thin section of the femur. (A) without and (B) with identification of the large ‘uniform’ regions. Color labels in (B) indicate: bone tissue and bone matrix (white), medullary and other cavities (ochre), cones of calcified cartilage (blue). Cavities are filled with sandy sediment of the matrix. The diaphysis is located to the left and the metaphysis to the right of the image.

The length of the preserved part of this femur is 22.3 mm and the bone shaft diameter varies between 7.5 and 8.0 mm. From a thin section in longitudinal view, three structures can be observed: the medullary cavity, the cortex and two cones of calcified cartilage (Fig 10).

The cortico-diaphyseal index (CDI) is about 2/3. Compared with other hadrosaurids, this ratio is consistent with that of embryos and hatchlings of other hadrosaurid species, such as *Hypacrosaurus stebingeri* [10] and *Maiasaura peeblesorum* [27]. Throughout ontogeny, this ratio gradually increases and adult hadrosaurids usually have a very large medullary cavity [27]. The medullary cavity is offset relative to the longitudinal axis of the femur. Because the femur was in a mediocre state of preservation, it could not be oriented unambiguously. Hence, the direction of the offset medullary cavity cannot be ascertained.

The earliest primary bone tissue is eroded away, creating a remarkably sharp (for a perinatal hadrosaurid) contact between the medullary cavity and the surrounding cortex (compare with, e.g., [10, 27, 28]). Such a sharp contact is most likely related to resorption of the cartilage precursor and contact with the initial bone tissues and the onset of the active expansion of the medullary cavity, although this is more common in larger (and older) specimens [27]. Scouring of the cortex by the sediment infill can be dismissed, because this would require a strong fluvial or aeolian action, which should also have resulted in a much high degree of disarticulation and transport of the bones, than is observed.

**Fig 11:**
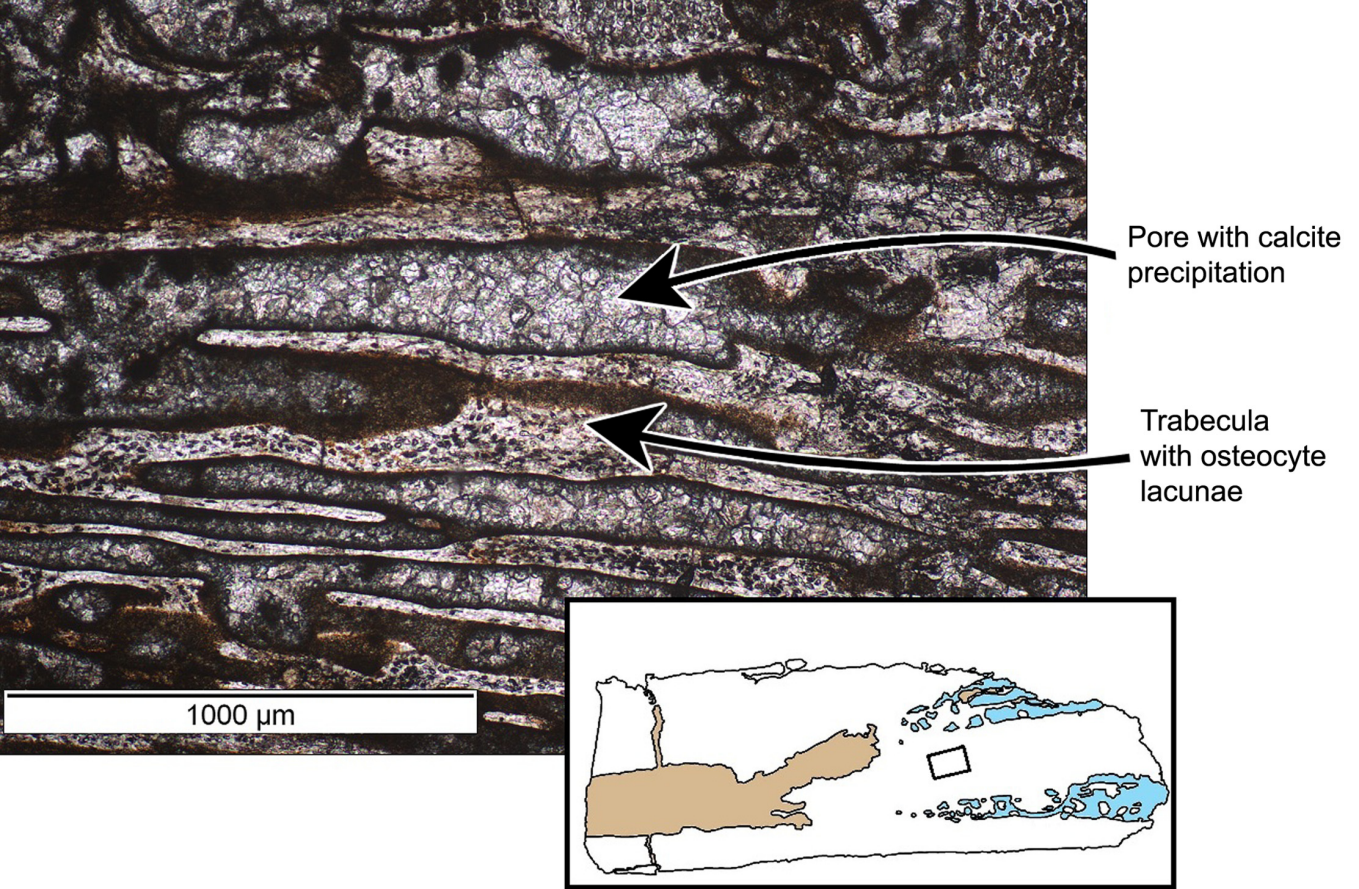
Perinatal specimens of *Saurolophus angustirostris* (MPC-D100/764). Woven bone structure (close-up of Fig 10), showing elongated but interconnecting trabeculae and pores with random distribution of osteocyte lacunae. Inset: location of the close-up within the bone, between two cones of calcified cartilage.

The cortex of the femur consists of a spongy network of primary trabeculae, as reflected by the high porosity of the bone (Fig 11). This network of bony struts creates a vascular network showing a varying degree of orientation, strong longitudinal orientation near the cones of calcified cartilage, and more plexiform orientation in the shaft.

The primary trabeculae contain numerous randomly oriented and irregularly-shaped osteocyte lacunae, indicative of woven bone (see [29, 30])(Fig 11).

**Fig 12:**
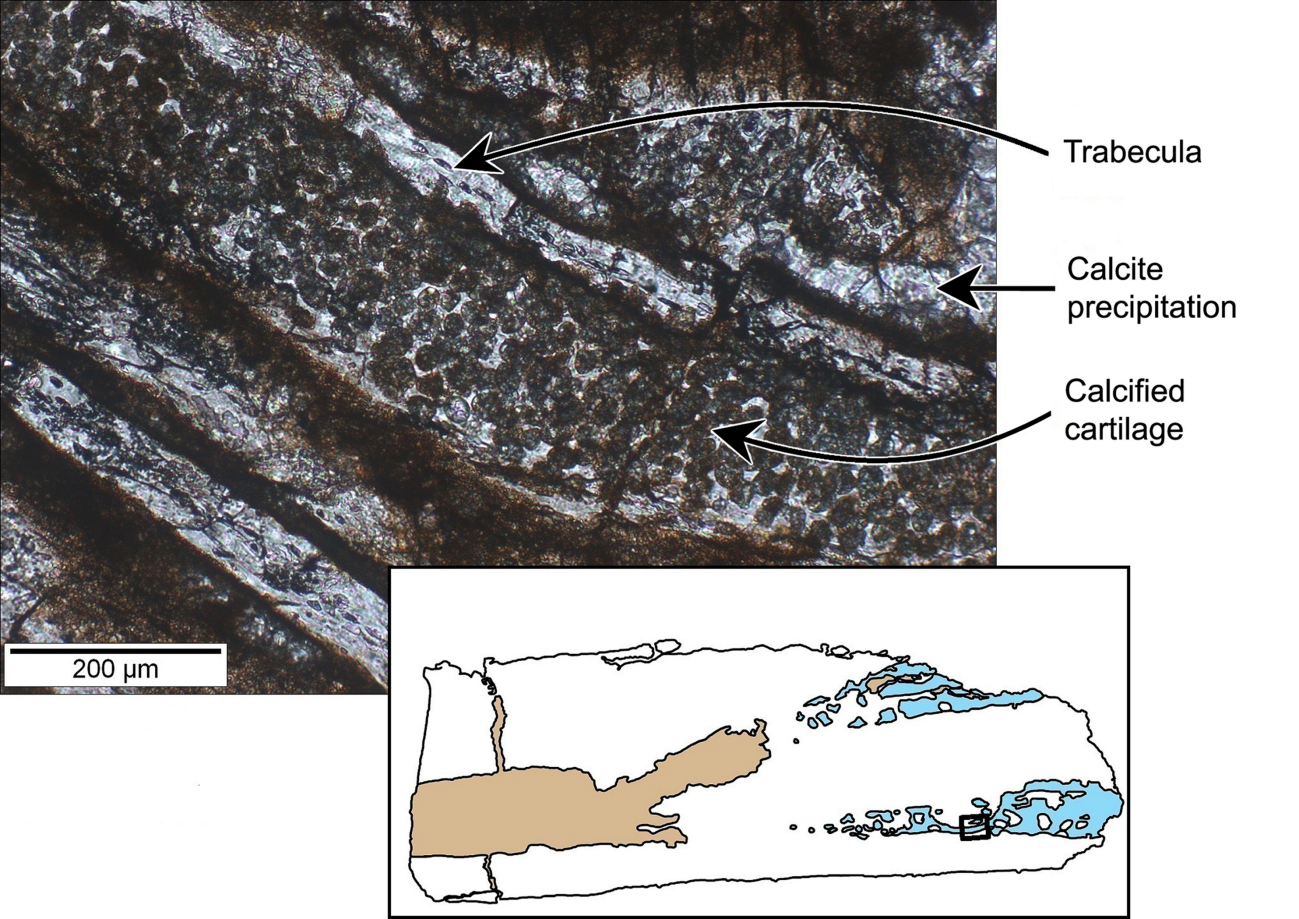
Perinatal specimens of *Saurolophus angustirostris* (MPC-D100/764). Calcified cartilage (close-up of Fig 10). Calcified cartilage preserved as small transparent and translucent globules with sizes in the order of a few tens of micrometers. Orange-brown matter is hematite dispersed within the cones of calcified cartilage, but especially concentrated at the contact surface between the calcified cartilage and the bone tissue. Inset: location of the close-up within the bone.

In the metaphyseal area of the diaphysis, the calcified cartilage cone is separated in two halves by a wedge of intramembraneously-formed bone tissues (Fig 10). The calcified cartilage is easily identified by the presence of small globules of calcite only a few tens of micrometers in diameter (Fig 12). These globules represent calcified chondrocytes and the space between the individual globules represents the calcified extracellular cartilage matrix. Many “islands” of endochondral bone tissue are dispersed throughout the cones of cartilage, showing active growth of bone, replacing the calcified cartilage. There are no signs of remodeling except for the resorption features in the medullary cavity and calcified cartilage cone.

### Vertebral histology

**Fig 13:**
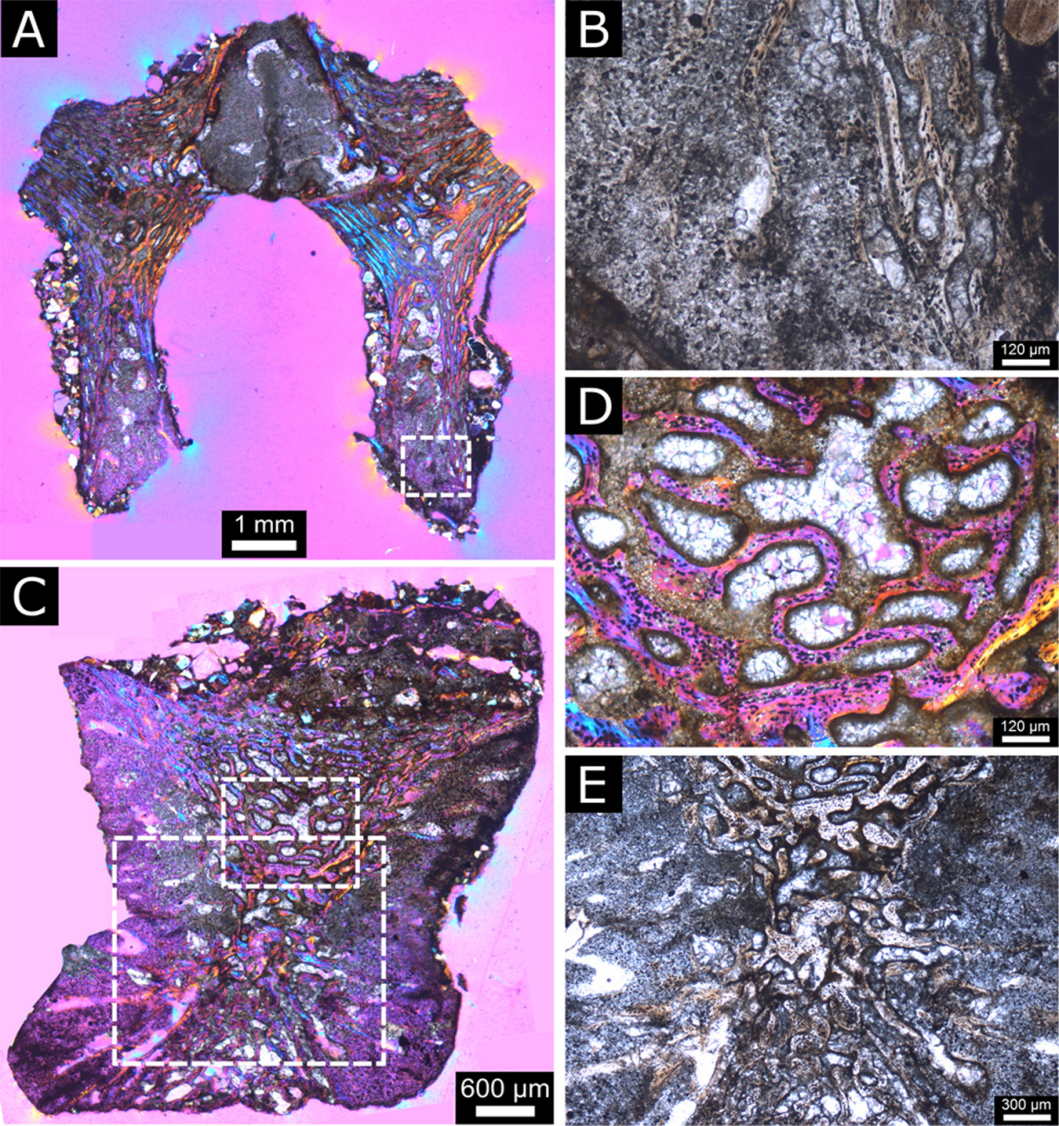
Perinatal specimens of *Saurolophus angustirostris*, dorsal vertebra histology. (A) Neural arch sectioned in anteroposterior plane. Note the dorsal fusion zone between the two somitic halves of the arch as well as the advanced state of ossification. (B) Enlarged view of boxed area in (A) showing preservation of the cartilaginous fusion zone between neural arch and vertebral body. (C) Vertebral body sectioned in the lateromedial plane. (D) Enlarged view of boxed area in (C) showing thin struts of woven bone trabeculae containing numerous osteocyte lacunae. (E) Enlarged view of boxed area in (C), detailing the absence of an open notochordal canal, and onset of ossification in the core of the vertebral centrum. (A), (C), (D) in cross polarized light with lambda waveplate filter, (B), (E) in plane polarized light.

The vertebral centrum measures 6.01 mm in width, 5.22 mm in length and 5.14 mm in height. The largely ossified neural arch measures 8.12 mm in width and 7.84 mm in height. In a craniocaudal section of the neural arch (Fig 13A and 13B) the fusion between—as well as the advanced ossification of—the two somitic halves is clearly visible. The pedicles of the arch largely consist of calcified cartilage and contain some endochondrally formed bone spicules (Fig 13B). In a lateromedial section of the vertebral centrum (Fig 13C, 13D and 13E), the advanced ossification pattern becomes clear. Thin primary trabeculae of woven bone (Fig 13D) comprises the main body of the centrum, and two cones of calcified cartilage containing large resorption spaces and struts of endochondral bone comprise the anterior and posterior zones of longitudinal growth with the articular surfaces. A notochordal canal is absent, however a central suture in the articular surfaces as well as newly formed woven bone trabeculae in the innermost core of the centrum (Fig 13E) indicate that the notochord has been completely resorbed.

## Eggshell Description and Identification

Two eggshell fragments were present on MPC-D100/764. These eggshell fragments were found at the articulated skeleton on the block (Fig 3), between the skull (Fig 4) and the hind limb (Fig 9). However, these eggshell fragments were removed from the block prior to photographing.

**Fig 14:**
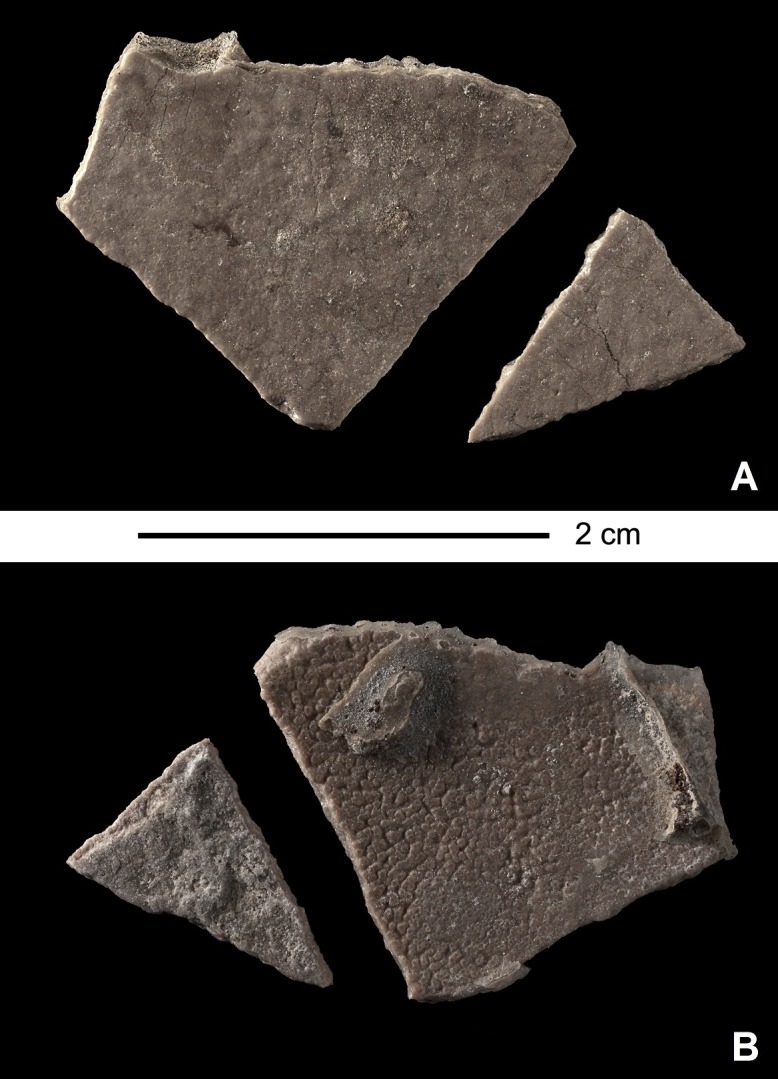
Perinatal specimens of *Saurolophus angustirostris* (MPC-D100/764). Macroscopic view of both eggshell fragments. (A) smooth or faintly sagenotuberculate outer surface and (B) mammillae on the inner surface.

The two eggshell fragments are very small (Fig 14). The larger fragment is trapezoidal, with a diagonal of 24 mm. The smaller fragment is triangular, less than 14mm long. Given the small size of the eggshell fragments, it is impossible to assess the size or shape of the entire egg.

Only faint nodes and ridges can be observed on the outer surface of the two eggshell fragments associated with the perinatal specimens, but these features are too poorly preserved for unambiguously distinguishing between ramotuberculate and sagenotuberculate ornamentations (Fig 14A). In any case, outer surface ornamentation is not a robust diagnostic character for identifying oospecies, particularly within the oogenus *Spheroolithus*, in which ramotuberculate, sagenotuberculate and smooth outer surfaces can be observed (e.g. [3, 31]). Moreover, *Spheroolithus* surface ornamentation can even differ significantly within a single egg [32].

**Fig 15:**
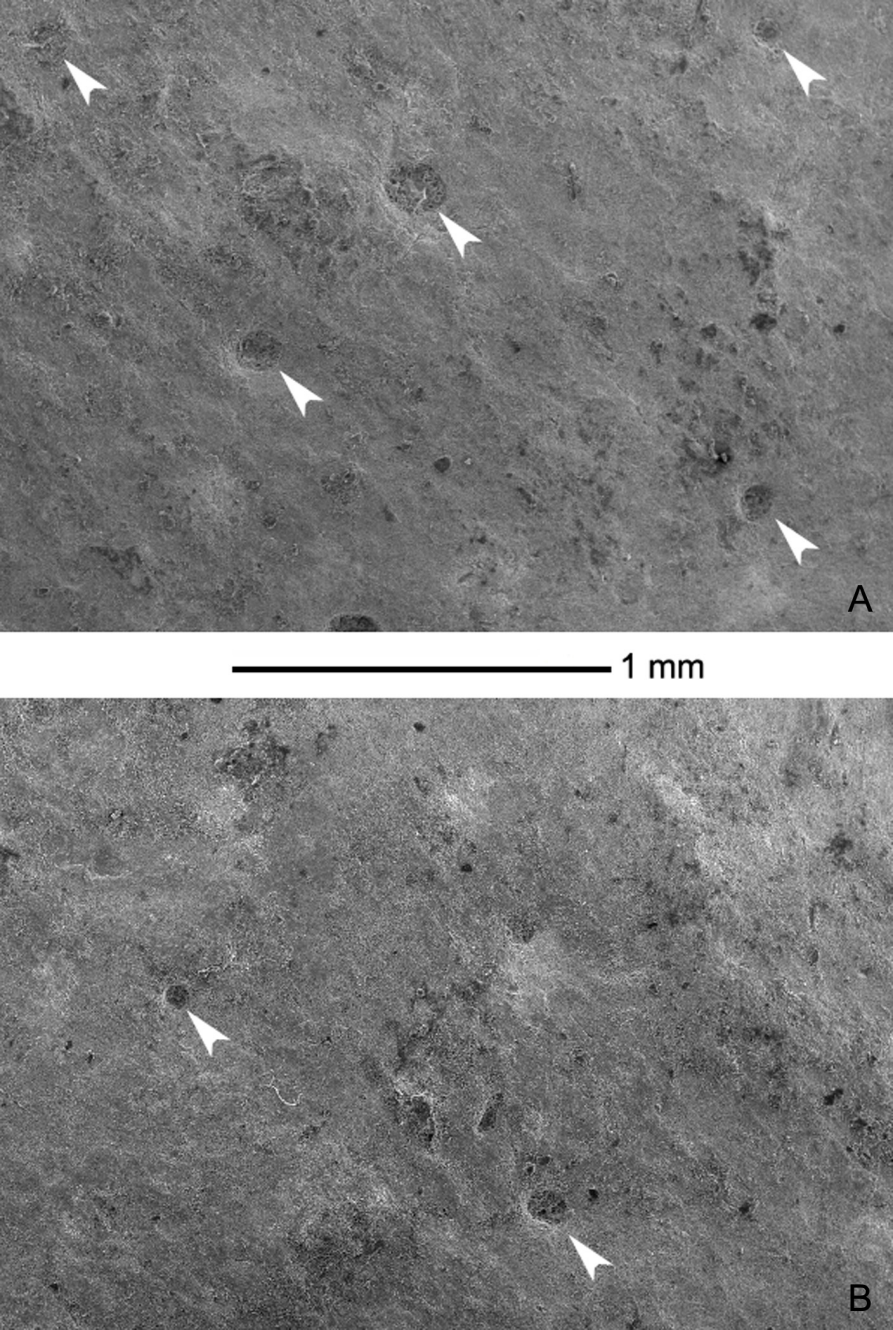
Perinatal specimens of *Saurolophus angustirostris* (MPC-D100/764). SEM photographs of the outer eggshell surface. (A) and (B) photographs of different parts of the outer eggshell surface. Low-relief pore openings indicated by white arrows.

Pore openings on the outer surface are widely spaced, with a density of 1–2 openings/mm^2^ and they do not show any organization (Fig 15). Pore openings are round to slightly oval in shape with diameters varying between 70 and 140 μm and very little relief. Many small cracks are also present on the outer surface of both eggshell fragments (Fig 14A).

**Fig 16:**
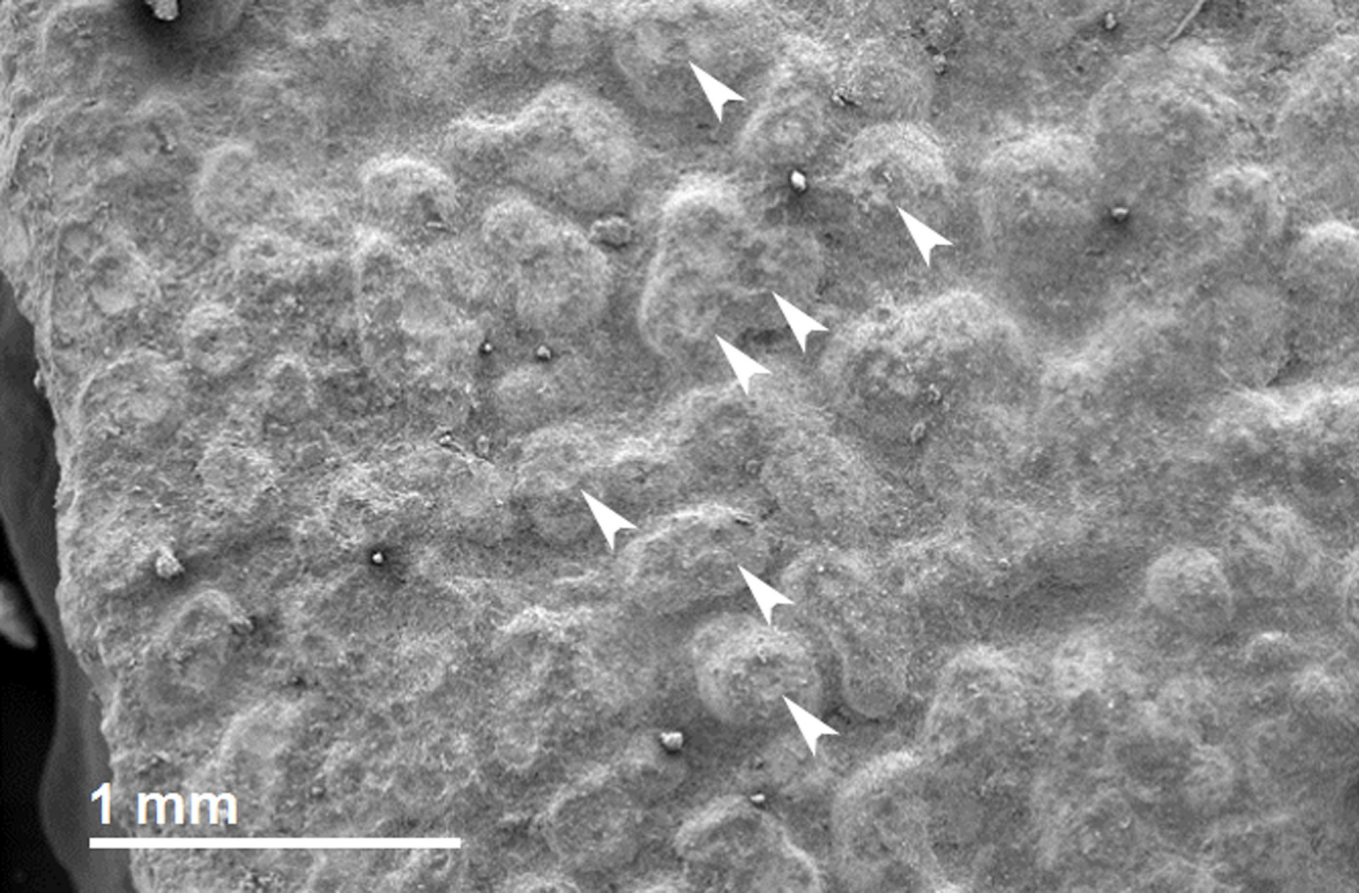
Perinatal specimens of *Saurolophus angustirostris* (MPC-D100/764). SEM photograph of mammillae. White arrows indicate a selection of the mammillae that are ‘cratered’. These ‘craters’ are most likely caused by calcite resorption by the individual, at the end of the embryonic stage.

**Fig 17:**
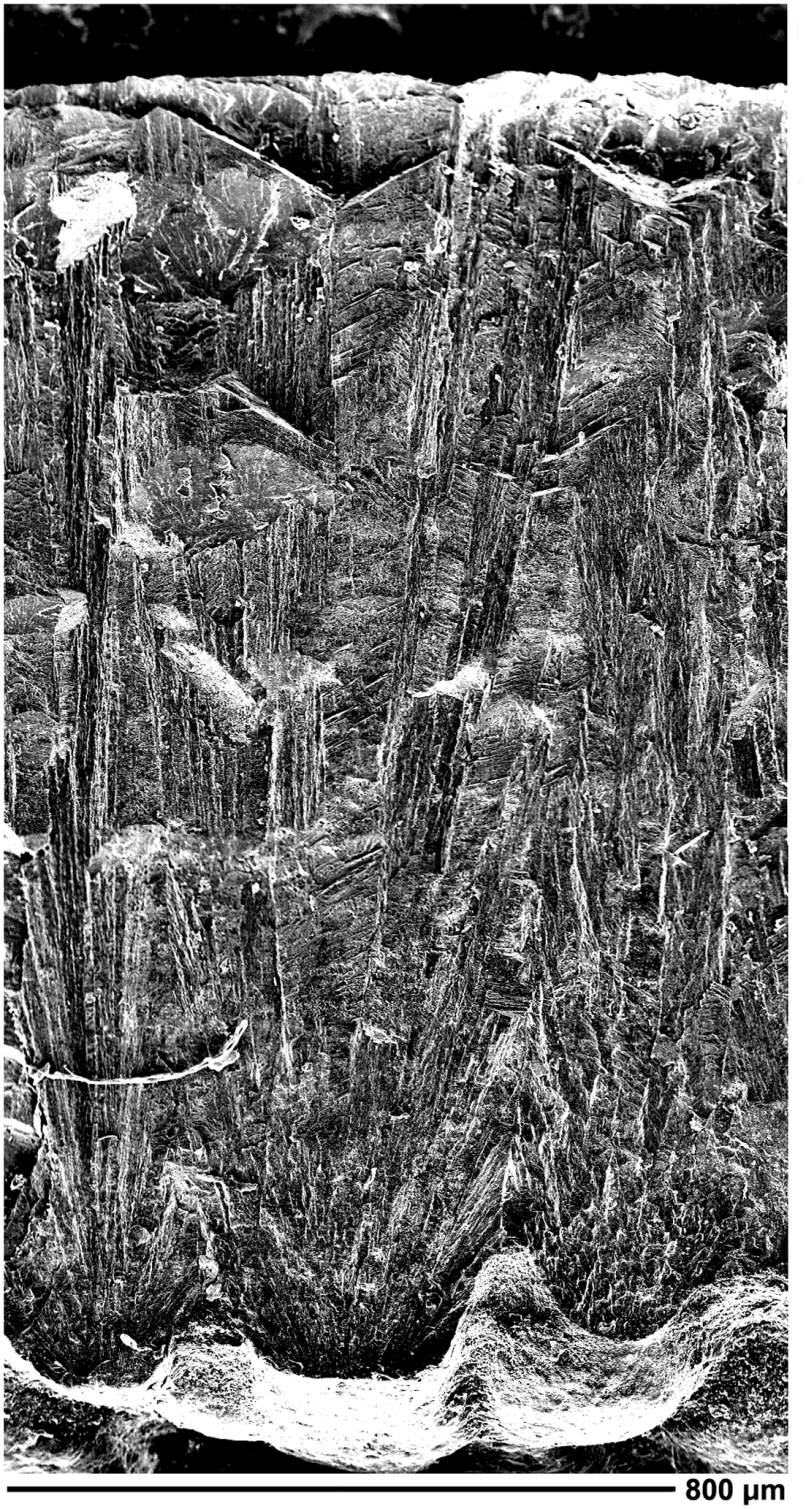
Perinatal specimens of *Saurolophus angustirostris* (MPC-D100/764). Sharpened composite SEM photograph of the dinosaurid-spherulithic ultrastructure of the eggshell. Eggshell units consist of calcite crystals radiating from the mammillae and exhibit a ‘herringbone’ pattern toward the outer surface of the eggshell units.

The inner surface is characterized by nearly circular to elliptical mammillae (Fig 14B). Most mammillae are pitted in their center, yielding a ‘crater-like’ appearance (Fig 16). The diameter (100–400 μm) and density (10–20/mm^2^) of these mammillae are locally variable, resembling the condition in *Spheroolithus irenensis* (12–21 mammillae/mm^2^) and contrasting with the less dense mammillae in *Spheroolithus chiangchiungtingensis* (7–14/mm^2^) [23]. No pore canals could be observed under SEM (Fig 17). Cratering and erosion of the mammillae may result from resorption of the calcite of the mammillae by the growing embryo, which needs calcium for bone growth [33]. This is in line with other observations (i.e., osteological anatomy and osteohistology) supporting the perinatal nature of the *Saurolophus angustirostris* babies.

Dissolution during diagenesis is unlikely because calcite resorption mainly led to the cratering of the mammillae. Diagenetic dissolution would rather result in overall smoothening of the inner surface of the mammillae. Nevertheless, the very limited number of pore canals observed (especially under SEM) indicates some degree of calcite dissolution and redeposition, rendering pore canals very hard to observe.

Furthermore, although it could not be falsified by direct observations, the predominantly round shapes of the pore openings on the outer surface and their sparse occurrence favor a non-branching angusticanaliculate, tubocanaliculate or prolatocanaliculate pore system. *Spheroolithus* is commonly associated with the latter pore system.

The eggshells have a prolatospherulitic morphotype, characteristic for the Spheroolithidae oofamily (e.g., [3, 31]) (Fig 14). The separate shell units are relatively loosely arranged at the inner margin of the eggshell, yet not easily separable toward the outer margin of the eggshell (Figs 14B and 17). For the inner zone of the shell, the individual adjacent shell units are visually distinct; while in the upper zone, adjacent shell units are visually inseparable. Yet, this does not imply the actual existence of two different zones. In (prolato)spherulitic eggshells, each eggshell unit remains distinct from the adjacent eggshell units along the thickness of the eggshell. The radial-tabular nature of the calcite crystals is clearly visible as a herringbone structure (Fig 17). The thickness of the eggshell fragments of this study is 1.80 +/- 0.11 millimeter, based on 10 measurements (S2 Table). This value lies well within the known thickness range in *Spheroolithus irenensis*, (1.4–2.0 mm; see Table 1), but contrasts with the thinner eggshells in *Spheroolithus tenuicorticus* (1.0–1.3 mm), *Spheroolithus albertensis* (1.0–1.5 mm) and *Spheroolithus maiasauroides* (1.3–1.5 mm). *Spheroolithus chiangchiungtingensis* (2.1–3.0 mm) and *Spheroolithus megadermus* (5.5–5.8 mm) have thicker eggshells (see [3]). However, these thickness ranges are the average ranges. True and complete thickness ranges may be considerably larger for individual ootaxa. For *Spheroolithus tenuicorticus*, the currently known absolute thickness interval ranges from 0.8 to 1.8 mm.

**Table 1:** List of all currently recognized *Spheroolithus* oospecies and their provenance.

*Spheroolithus* oospecies	Stratigraphic and geological setting	Age	Eggshell thickness (mean range)	References
*Spheroolithus albertensis*	Oldman Formation (Alberta, Canada); Two Medicine River Formation (Montana, USA)	Campanian	1.0–1.5 mm	[34]
*Spheroolithus chiangchiungtingensis*	Wangshi Group (Shandong, China)	Upper Cretaceous	2.1–3.0 mm	[3, 35]
*Spheroolithus irenensis* (= *Paraspheroolithus irenensis*)	Wangshi Group (Shandong, China); Iren Dabasu Formation (Inner Mongolia, China); Nemegt Formation (Mongolia)	Upper Cretaceous; late Campanian—early Maastrichtian	1.1–2.2 mm (1.4–2.0 mm)	[3, 35–39]
*Spheroolithus maiasauroides*	Djadokhta Formation (Mongolia)	Campanian	1.0–1.6 mm (1.2–1.5 mm)	[3, 38, 39]
*Spheroolithus megadermus*	Wangshi Group (Shandong, China)	Upper Cretaceous	5.0–6.0 mm (5.5–5.8 mm)	[3, 23, 36]
*Spheroolithus tenuicorticus*	Barun Goyot Formation, Mongolia	Campanian	0.8–1.8 mm (1.0–1.3 mm)	[3, 23, 38]

Amongst the known ootaxa, the eggshells associated to the perinatal *Saurolophus angustirostris* specimens most closely resemble *Spheroolithus irenensis*. Eggshells of the dinosaurid-spherulithic type are usually attributed to ornithischian or sauropod dinosaurs [40]. Among the dinosaurid-spherulithic eggs, the oofamily Spheroolithidae had been proven to be associated with ornithopods, and probably exclusively with hadrosauroids [40–42]. *Spheroolithus* is exclusively known from the Upper Cretaceous (Table 1). *Spheroolithus albertensis* is the only North American *Spheroolithus* oospecies and has been collected from a nesting site containing *Maiasaura* babies [34, 41]. Spheroolithid eggshells have also been found associated with the lambeosaurine *Hypacrosaurus stebingeri* [10].

All the other currently recognized *Spheroolithus* oospecies are restricted to Eastern Asia. *Spheroolithus irenensis* has been found associated with skeletal remains referred to the hadrosauroid *Bactrosaurus*, in Laiyang (Shandong Province, China; [35]). However, the identification of the Laiyang skeletal elements needs to be confirmed. Moreover, the taxonomical history of *Spheroolithus irenensis* is particularly confused. Originally, Young [36] erected the ootaxon *Oolithus spheroides*. In a first revision, Zhao and Jiang [35] separated eggs from this taxon into three different new taxa: *Spheroolithus irenensis*, *Spheroolithus chiangchiungtingensis* and *Spheroolithus chingkangkouensis*. Later, Zhao [37] reassigned these three species to three separate genera: *Paraspheroolithus irenensis* for *S*. *irenenis*, *Ovaloolithus chinkangkouensis* for *Spheroolithus chingkangkouensis*, whereas *Spheroolithus chianchiungtingensis* remained unchanged. In a later study, Mikhailov [3] considered *Paraspheroolithus* as a junior synonym of *Spheroolithus*. Currently, there is still no unanimity about the position of the species. Although many researchers consider the species to belong to *Spheroolithus* [3, 23, 39], others still consider that it belongs to *Paraspheroolithus* [43].

*Spheroolithus chiangchiungtingensis* and *Spheroolithus megadermus* are exclusively known from the Wangshi Group in Shandong Province, China. *Spheroolithus maiasauroides* was described from the late Campanian Djadokhta Formation and *Spheroolithus tenuicorticus* from the Barun Goyot Formation, which underlies the Nemegt Formation in the Nemegt Basin. Carpenter [23] considers that *Spheroolithus irenensis* and *Spheroolithus tenuicorticus* are synonymous. With the current limited number of eggshell fragments of *Spheroolithus tenuicorticus*, the only known difference between both oospecies is the eggshell thickness [3, 23].

## Discussion

### Ontogenetic stage of the perinatal specimens

Multiple features indicate that the *Saurolophus angustirostris* specimens studied in this paper are perinatal individuals, but do not help in determining whether the individuals are still embryonic or neopionic (postembryonic) when they died.

The skull length is estimated to be in the order of six centimeters (Fig 3), about five percent of the skull length of the largest known individual of *Saurolophus angustirostris* (1220 mm in PIN 551/357). It is about the same size as the skull of embryonic specimens in the lambeosaurine *Hypacrosaurus stebingeri* [10].

Some bones are obviously spongy at larger magnification (Fig 6), indicating that bone growth is still dominated by growth of trabeculae and woven bone. This corresponds with histological observations in the section of the distal femur and dorsal vertebra described above. The predominance of primary trabecular woven bone is indicative of rapid bone growth, a characteristic for embryos, hatchlings and juveniles (e.g., [27, 28]). The complete closure of the notochordal canal may indicate a postembryonic stage (for saurischians, see [44, 45]), however it remains unclear if vertebral development in ornithischian and saurischian dinosaurs followed the same timing and ossification patterns. Therefore, these observations do not aid in the unequivocal differentiation between a late embryonic or hatchling stage for MPC-D100/764, but give a preliminary indication of a postembryonic stage, in the absence of further osteohistological studies of perinatal hadrosaurid vertebrae.

The poor preservation of the extremities of the long bones testifies for their poor ossification. Poor ossification of the joints is also observed in many other dinosaurian embryos and (possibly altricial) hatchlings (e.g., [27, 28]).

Few vertebral centra are associated with their neural arches and neural spines. This is especially true for the caudal vertebrae of the partially articulated skeleton, where no neural arches are present at all (Fig 9). Thus, contact of the vertebral centra with their associated neural arches might have been very weak or absent at the time death of the individuals of this specimen. However, fusion between the neural centra and their associated neural arches usually occurs relatively late in the ontogeny of archosaurs (see [46]). Unfused halves of neural arches also reveal a very early developmental stage, as it has only been observed in an embryonic *Camptosaurus* specimen, in dinosaurs [22]. Hence, *Saurolophus angustirostris* is only the second dinosaur species with perinatal specimens actually showing unfused neural arches. Consequently, the observation of unfused neural arches is strongly supportive of an embryonic stage for MPC-D100/764, because many other hadrosaurids such as *Hypacrosaurus* [10] and *Maiasaura* [47] already show fused neural arches while still in the embryonic stage.

The presence of a relatively narrow medullary cavity in cross-section with resorption of the cortex indicates that the studied individuals experienced maximal rates of bone growth and remodeling and, hence, that they were in the embryonic or hatchling stage (e.g., [9, 27]).

The association of two eggshell fragments with small *Saurolophus angustirostris* individuals suggests that they belong to the eggs that contained the perinatal individuals. Together with the cratering of many of the cones on the internal surface of the eggshell, this strongly suggests that the eggshell fragments stem from a near-term egg or an egg from a nestling that already hatched [23, 33].

### Early ontogeny in *Saurolophus angustirostris*

The specimens in the MPC-D100/764 display several features, widely distributed amongst terrestrial tetrapods and reflecting their earliest ontogenetic stages: a proportionally large skull, large orbits, a proportionally robust postcranium, and unfused neural arches and centra.

Ontogenetic changes within *Saurolophus angustirostris* have already been described by Rozhdestvensky [48], Maryańska and Osmólska [11], and then by Bell [8], on the basis of an ontogenetic series including juveniles, subadults and adults. For the first time, we are now able to reconstruct the morphological changes that took place during the early ontogeny of a saurolophine dinosaur. During development from the perinatal stage to the juvenile stage, the snout became proportionally longer, the orbit became more elongated dorsoventrally and inclined caudodorsally, the doming of the frontal became less prominent, and the coronoid process stood up straighter, perpendicular to the mandibular axis. Those changes continued throughout more advanced ontogenetic stages, from the juvenile to the adult stages ([11]; Table 2). Ontogenetic changes described by Bell [8] on the lateral wall of the braincase could not be adequately observed in the perinatal specimens, due to poor preservation of the braincase.

**Table 2:** Cranial ontogenetical changes in *Saurolophus angustirostris*, as listed by Maryanska and Osmolska [1 1], with incorporation of observations on the hatchling specimens.

Characters as listed by Maryańska and Osmóslka [11]	In MPC-D100/764	Remarks
Snout becoming longer with age	Observed	Very small snout
Posterior declination of long axes of orbit and infratemporal fenestra with age	Orbit observed; infratemporal fenestra not preserved	Infratemporal fenestra not adequately preserved
Doming skull flattening with age	Observed	Strong convexity frontals
Contribution of parietal in anterior margin of temporal fenestra increases with age	Not observed	Skull roof not adequately preserved
Decrease and dissapearance of parietal separating squamosal	Not observed	Skull roof not adequately preserved
Supraoccipital becoming more horizontal with age	Not observed	Occiput not adequately preserved
Longitudinal ridge on dorsocranial surface nasal becomes higher with age	Not observed	Nasal not adequately preserved
Nasal gradually overhangs the external naris dorsally with age.	Not observed	Naris not observed
Jugal—lacrimal contact becomes thicker with age	Not observed	Possible post-mortem deformation bones
Dental battery less than half length mandible in young becoming longer with age	Not observed	Dental battery embedded in host rock
Coronoid process perpendicular to mandibular axis becoming slightly acute with age	Observed	Angle even slightly obtuse in hatchling
Predentary opens dorsally with age	Not observed	Predentary too much embedded in host rock

Additional changes that occured early in ontogeny and could not be observed in later development stages concern the development of the supracranial crest and the fusion of the left and right halves of the neural arches.

Nasal crest structures are obviously absent in the earliest developmental stages in *Saurolophus angustirostris*, even thought it cannot be completely excluded that taphonomic processes explain this absence. However the crest in the latter stages of *Saurolophus* is particularly robust [8], so it is unlikely that the crest would have been broken off and removed taphonomically, while more fragile and slender skull bones are still present and particularly well preserved. The supracranial crest is also absent in the youngest lambeosaurine specimens, including *Lambeosaurus* [49], *Hypacrosaurus* [10, 16], *Corythosaurus* [15], and *Parasaurolophus* [50]. It must be noted that the crest becomes proportionally larger throughout later ontogenetic stages, being particularly small in the juvenile ZPAL MgD-1/159 then progressively larger in subadults (PIN 551/356) and adults (MPC 100/706 and PIN 551/357). However, the ambiguous incipient bifurcation of the frontal into a caudodorsal and a rostroventral process might indicate the absence of a supracranial crest, or the existence of only an indistinctly small crest in the perinatal specimen, similar to the very small rounded nasal crest observed in the juvenile lambeosaurine *Kazaklambia convincens* [17].

The separation between the left and right halves of the neural arches, present on some cervical vertebrae of the perinatal specimens, has never been documented in hadrosaurids before. In the embryonic development of mammals [51], two neural processes develop around the spinal cord; these neural processes subsequently fuse dorsally to the spinal cord and form a neural arch that grows dorsally to form the neural spine.

## Conclusions

MPC-D100/764 represents part of a nest of perinatal hadrosaurids. The eggs were originally laid on a point bar along a riverbank. Whether the individuals are still embryonic or neopionic (postembryonic) when they died cannot be accurately determined. The babies were apparently already dead and partly decomposed when they were buried by sediment entrained by the river current during the wet summer season. Coincidence of hatching and the wet summer season has widely been assumed but rarely been observed among hadrosaurids.

The babies already displayed diagnostic characters for *Saurolophus angustirostris*, including premaxillae with strongly reflected oral margin and an upturned premaxillary body in lateral aspect. They represent the earliest ontogenetic stages known for this species and thus bridge a large gap in our knowledge of the ontogeny of *S*. *angustirostris*. The absence of a supracranial crest and unfused halves of the cervical neural arches characterize the earliest stages in the ontogeny of *S*. *angustirostris*. The eggshell fragments associated with the perinatal individuals can be referred to as the *Spheroolithus* oogenus and closely resemble those found in older formation (e.g. Barun Goyot Fm in Mongolia) or associated with more basal hadrosauroids (*Bactrosaurus*-*Gilmoreosaurus* in the Iren Dabasu Fm, Inner Mongolia, China). This observation suggests that the egg microstructure did not evolve significantly during the last stages of the hadrosauroid evolution.

## Supporting Information

S1 File Excel dataset of grain size analysis generated by ImageJ software. (XLS)

S1 Table Length measurements of MPC-D100/764. (DOCX)

S2 Table Thickness measurements of eggshell fragments of MPC-D100/764. (DOCX)

## Abbreviations

MPC: Institute of Paleontology and Geology, Mongolian Academy of Sciences, Ulaan Baatar, Mongolia
PIN: Paleontologiceski Institut, Academii Nauk, Moscow, Russia
RBINS: Royal Belgian Institute of Natural Sciences, Brussels, Belgium
ZPAL: Institute of Paleobiology of the Polish Academy of Sciences, Warsaw, Poland
