# Supplementary material for: Perinatal Specimens of Saurolophus angustirostris (Dinosauria: Hadrosauridae), from the Upper Cretaceous of Mongolia
Source: PLoS One. 2015 Oct 14;10(10):e0138806. doi: 10.1371/journal.pone.0138806 (PMC4605499; doi:10.1371/journal.pone.0138806)
Supplement: S1 Table — (DOCX) [file pone.0138806.s002.docx]

**Supporting Information**

**S1 Table. Length measurements of MPC-D100/764.**

The number of length measurements of MPC-D100/764 is strongly limited by the following characteristics: 1) bones are often severely fractured or broken and, hence, incomplete; 2) given the perinatal ontogenetic stage of the bones, many bones most likely were not completely ossified at the time of death of the individuals; 3) it is impossible to retrace the exact position of each vertebra, hence, length measurements of vertebrae are otiose.

When measured bones are very incomplete, the minimal length is indicated by “>”.

Length measurements were taken with a Vernier caliper to 0.1mm.

|  | **Length (0.1 mm)** |
| --- | --- |
| **Maxilla ventral length** | 20.6 |
| **Jugal length** | >20.2 |
| **Total length articulated skull (premaxilla – preserved part of jugal)** | 49.1 |
| **Anteroposterior length mandible** | >33.1 |
| **Humerus length** | 31.8 |
| **Articulated (disarticulated) femur length** | >33.7 (>43.5) |
| **Articulated (disarticulated) femur width diaphysis** | 6.3 (6.8) |
| **Articulated (disarticulated femur width distal epiphysis** | 8.8 (8.7) |
| **Length prepubis** | 12.8 |
| **Articulated tibia length** | 37.1 |
| **Fibula length** | >34.2 |
| **Fibula width diaphysis** | 2.0 |
| **Fibula width distal epiphysis** | 4.2 |
| **Length metatarsal II** | 10.7 |
| **Length metatarsal III** | 11.7 |
| **Length metatarsal IV** | 9.6 |
