# Supplementary material for: Perinatal Specimens of Saurolophus angustirostris (Dinosauria: Hadrosauridae), from the Upper Cretaceous of Mongolia
Source: PLoS One. 2015 Oct 14;10(10):e0138806. doi: 10.1371/journal.pone.0138806 (PMC4605499; doi:10.1371/journal.pone.0138806)
Supplement: S2 Table — (DOCX) [file pone.0138806.s003.docx]

**Supporting Information**

**S2 Table. Thickness measurements of eggshell fragments of MPC-D100/764.**

|  | **Thickness (0.01mm)** |
| --- | --- |
| **1** | 1.80 |
| **2** | 1.76 |
| **3** | 1.87 |
| **4** | 1.75 |
| **5** | 2.00 |
| **6** | 1.80 |
| **7** | 1.73 |
| **8** | 1.62 |
| **9** | 1.72 |
| **10** | 1.95 |
| **total** | **1.80** |
| **error** | **0.11** |
